# METTL3-mediated m^6^A modification of DNMT1 enhances ovarian cancer progression

**DOI:** 10.1126/sciadv.aec2297

**Published:** 2026-11-04

**Authors:** Kanchan Kumari, Iván Pérez-Núñez, Aleix Noguera Castells, Raquel Pluvinet, Carlos Peula, Annika Patthey, Ghazala Naz Khan, Kinga Pajdzik, Ángel-Carlos Román, Erik Dassi, Chuan He, Eva Lundin, Manel Esteller, Toni Celià-Terrassa, Francesca Aguilo

**Affiliations:** ^1^ Department of Molecular Biology, Umeå University, SE-901 85 Umeå, Sweden.; ^2^ Wallenberg Centre for Molecular Medicine, Umeå University, SE-901 85 Umeå, Sweden.; ^3^ Cancer Research Program, Hospital del Mar Medical Research Institute (HMRI), Barcelona, Spain.; ^4^ Cancer Epigenetics Group, Josep Carreras Leukaemia Research Institute (IJC), Badalona, Barcelona, Catalonia, Spain.; ^5^ Department of Biosciences, Faculty of Science, Technology and Engineering, University of Vic-Central University of Catalonia (UVic-UCC), Vic, Barcelona, Catalonia, Spain.; ^6^ Centro de Investigacion Biomedica en Red Cancer (CIBERONC), Madrid, Spain.; ^7^ Genomics Unit, Josep Carreras Leukaemia Research Institute (IJC), Badalona, Barcelona, Catalonia, Spain.; ^8^ Department of Cellular, Computational and Integrative Biology (CIBIO), University of Trento, 38123 Trento, Italy.; ^9^ Department of Medical Biosciences, Umeå University, SE-901 85 Umeå, Sweden.; ^10^ Department of Chemistry, The University of Chicago, Chicago, IL 60637, USA.; ^11^ Department of Molecular Biology and Genetics, University of Extremadura, 06071 Badajoz, Spain.; ^12^ Cancer Epigenetics Group, Sant Pau Research Institute (IRSantPau), Barcelona, Catalonia, Spain.; ^13^ Institucio Catalana de Recerca i Estudis Avançats (ICREA), Barcelona, Catalonia, Spain.; ^14^ Physiological Sciences Department, School of Medicine and Health Sciences, University of Barcelona (UB), Barcelona, Catalonia, Spain.

## Abstract

High-grade serous ovarian carcinoma (HGSOC), the most lethal subtype of ovarian cancer, is often diagnosed at advanced stages and remains associated with poor clinical outcome. In this study, we identify a critical oncogenic role of the RNA methyltransferase METTL3 and the *N*^6^-methyladenosine (m^6^A) RNA modification pathway in HGSOC. Suppression of METTL3, either genetically or pharmacologically, and depletion of its binding partner METTL14 impair ovarian cancer proliferation and tumor progression. Mechanistically, we show that METTL3-mediated m^6^A deposition enhances the translation of DNA methyltransferase 1 (DNMT1), the primary maintenance DNA methyltransferase responsible for preserving genome-wide methylation patterns and silencing tumor suppressor genes. Pharmacological inhibition of DNMT1 induces widespread DNA hypomethylation and up-regulation of the tumor suppressors TNFAIP3 and FBXO32. Consistently, METTL3 suppression reduces DNMT1 abundance and derepresses TNFAIP3 and FBXO32 expression. Knockdown and rescue studies further reveal that DNMT1 primarily mediates METTL3-dependent regulation of cell migration, whereas the antiproliferative effects of METTL3 suppression are likely driven by additional downstream targets. Collectively, these findings identify the m^6^A-METTL3-DNMT1 axis as a mechanistic link between RNA and DNA methylation pathways and a potential therapeutic vulnerability in HGSOC.

## INTRODUCTION

Ovarian cancer is the most lethal gynecological malignancy and the fifth leading cause of cancer-related mortality among women (*1*). Ovarian carcinomas comprise several histological subtypes (*2*), among which high-grade serous ovarian carcinoma (HGSOC) is the most prevalent and aggressive. HGSOC is frequently diagnosed at advanced stages, often presenting widespread metastasis (*3*), highlighting the need to elucidate the molecular mechanisms driving its progression.

Beyond genetic alterations, epigenetic and epitranscriptomic mechanisms profoundly influence cancer phenotypes, including ovarian cancer (*4*–*6*). DNA methylation [5-methylcytosine (5mC)] represents a major epigenetic layer of gene expression control, playing crucial roles in genomic imprinting, X chromosome inactivation, and transposon repression (*7*). DNMT1, the primary maintenance methyltransferase, preserves methylation patterns during replication and sustains repressive chromatin landscapes (*8*). In ovarian cancer, DNMT1 promotes immune evasion by cooperating with EZH2 to silence T helper 1 (T_H_1)–type chemokines CXCL9 and CXCL10, thereby limiting effector T cell infiltration into the tumor microenvironment, a phenotype that can be reversed by epigenetic inhibitors (*9*). DNMT1 also contributes to chemoresistance by repressing the tumor suppressor RGS10 in conjunction with HDAC1 (*10*). Furthermore, dual inhibition of DNMT1 with HDAC6 enhances interferon signaling, restores antitumor immune responses, and reduces tumor burden in preclinical ovarian cancer models (*11*). Despite clear evidence of DNMT1 dysregulation in ovarian cancer, the upstream regulatory mechanisms governing its activity in HGSOC remain poorly characterized.

RNA modifications provide an additional regulatory layer of gene expression regulation. Among them, *N*^6^-methyladenosine (m^6^A) is the most abundant internal mRNA modification and modulates RNA stability, splicing, and translation. m^6^A is catalyzed by a methyltransferase complex containing METTL3, the catalytic subunit, and METTL14, along with other proteins such as WTAP, RBM15, VIRMA, and CBLL1, collectively referred to as “writers.” m^6^A is a reversible mark that can be removed by demethylases, known as “erasers,” such as “fat mass and obesity-associated” gene (FTO) and ALKBH5 (*12*), and recognized by “readers” including YTHDF1/2/3, YTHDC1/2, IGF2BP1/2/3, and HNRNPA2B1, which direct the fate of the modified transcripts (*13*). METTL3 has emerged as a prominent oncogene in ovarian cancer. METTL3 is consistently up-regulated in ovarian tumors (*14*, *15*). Functional studies have demonstrated that METTL3 promotes oncogenesis by activating the PI3K/AKT/mTOR pathway via miR-126-5p–mediated PTEN inhibition (*14*), enhancing AXL translation, and stabilizing oncogenic RNAs including *MALAT1*, *DDR2*, and *RHPN1-AS1* via m^6^A modification (*15*–*18*). METTL3 also regulates miR-1246 to repress CCNG2, thereby supporting tumor metabolism and proliferation (*19*). In contrast, the role of METTL14 in ovarian cancer remains ambiguous, with conflicting studies suggesting both tumor-promoting and tumor-suppressive effects (*20*, *21*).

We interrogated the roles of METTL3 and METTL14 in HGSOC. Depletion or pharmacological inhibition of METTL3, as well as depletion of its binding partner METTL14, impaired tumor cell progression and led to widespread deregulation of oncogenic pathways. m^6^A profiling identified *DNMT1* as an m^6^A-modified target. Although *DNMT1* transcript levels were unchanged, METTL3 depletion substantially reduced DNMT1 protein abundance through impaired translation. Functional analyses revealed that DNMT1 acts downstream of METTL3 to repress tumor suppressor genes, including TNFAIP3 and FBXO32, and to promote cancer cell migration. Together, these findings identify DNMT1 as a translationally regulated target of METTL3 and uncover a METTL3-DNMT1 regulatory axis that enhances HGSOC progression, pointing to a promising therapeutic vulnerability.

## RESULTS

### METTL3 and METTL14 enhance ovarian tumorigenesis

To investigate the role of METTL3 and METTL14 in ovarian cancer, we first performed knockdown experiments in the HGSOC OVCAR8 cell line using short hairpin RNAs (shRNAs) (referred to as sh1 and sh2 for each gene) along with a scrambled (Scr) control. Knockdown efficiency was validated by Western blot and reverse transcription quantitative polymerase chain reaction (RT-qPCR) (fig. S1, A to D). The depletion of either METTL3 or METTL14 substantially reduced cell proliferation (Fig. 1, A and B), colony formation (Fig. 1, C to E), and migration (Fig. 1, F to H). Annexin V staining revealed increased apoptosis (Fig. 1, I and J), whereas cell cycle analysis showed G_0_-G_1_ arrest and reduced G_2_-M populations (Fig. 1, K and L) in METTL3- and METTL14-depleted cells. Pharmacological inhibition of METTL3 using STM2457 elicited comparable, albeit milder, effects, consistent with the notion that METTL3 can also promote tumorigenesis through m^6^A-independent mechanisms distinct from its methyltransferase activity (*22*). STM2457 treatment impaired OVCAR8 cell proliferation, colony formation, and migration (fig. S1, E to J), whereas the increase in apoptotic cells was not statistically significant (fig. S1K). In addition, STM2457 altered cell cycle progression, reducing the proportion of cells in the G_0_-G_1_ and G_2_-M phases while increasing the S-phase population (fig. S1L). Notably, METTL3 silencing reduced METTL14 protein levels without affecting its mRNA levels, whereas METTL14 knockdown similarly decreased METTL3 protein abundance (figs. S1, A and B, and S2, A to C), suggesting mutual posttranscriptional stabilization, as previously observed in other cellular models (*23*, *24*). Supporting this, METTL14 was detected by immunoprecipitation of endogenous METTL3 (fig. S2D). To further investigate whether METTL3 stabilizes METTL14 in ovarian cancer, we generated doxycycline-inducible, shRNA-resistant, FLAG-tagged METTL3 in OVCAR8 cells. Reexpression of METTL3 restored the reduced METTL14 levels caused by METTL3 knockdown (fig. S2E). Moreover, treatment of cells with the proteasome inhibitor MG132 increased METTL14 protein levels in METTL3 knockdown cells (fig. S2, F and G), indicating that METTL14 undergoes proteasomal degradation in METTL3-deficient cells, as previously suggested (*23*). METTL3 depletion also lowered the protein levels of other writer complex components, such as WTAP and VIRMA, but not HAKAI, underscoring the role of METTL3 in preserving the integrity of the m^6^A methyltransferase complex (fig. S2H).

**Fig. 1.**
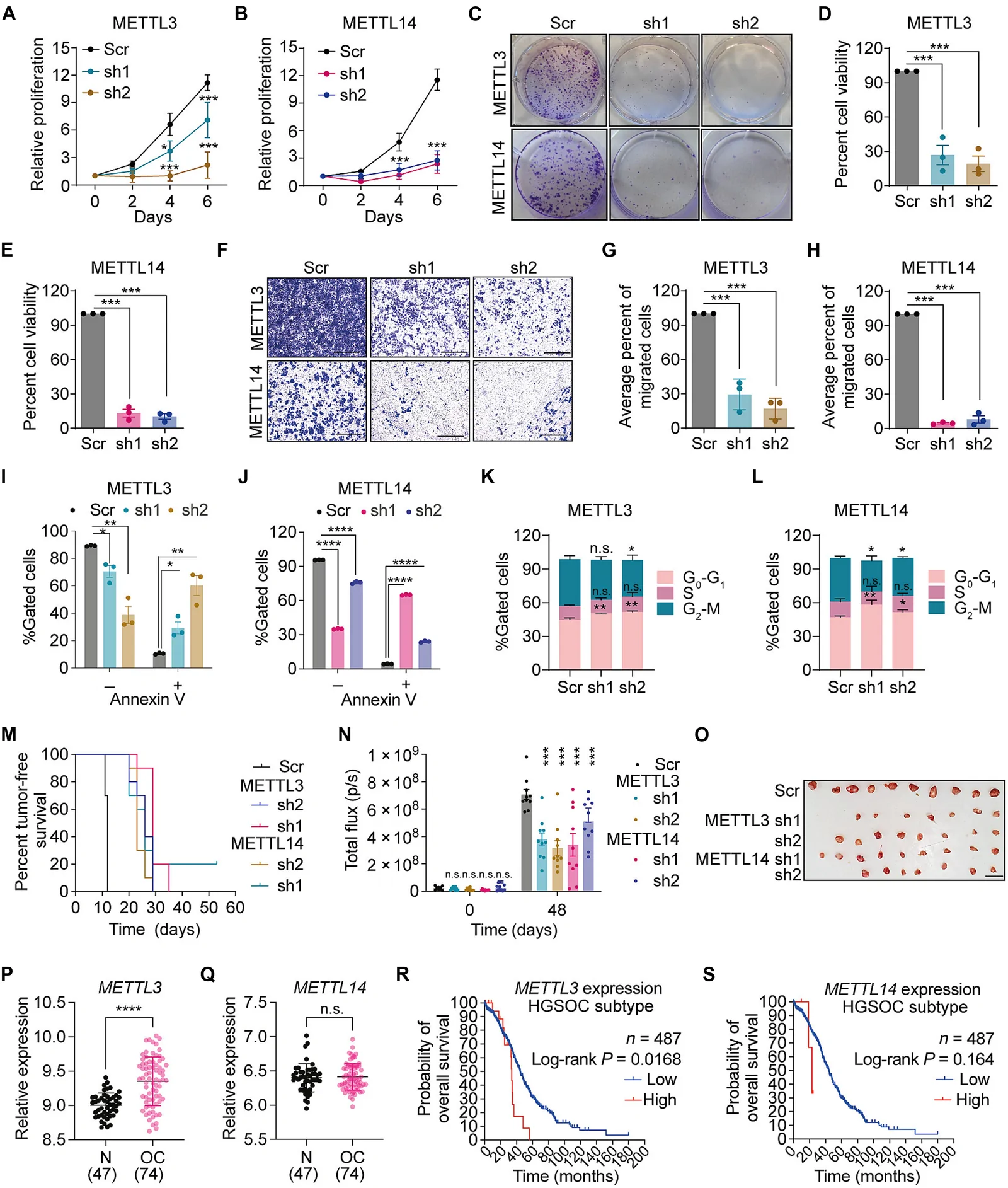
METTL3 and METTL14 promote ovarian cancer progression and associate with poor clinical outcome. (**A** and **B**) Cell proliferation rates of METTL3-depleted (A) and METTL14-depleted (B) cells measured over 6 days. (**C**) Representative colony formation assay images of METTL3- and METTL14-depleted cells over 10 days. (**D** and **E**) Bar graphs of the relative cell viability from the colony formation assay in (C), measured by crystal violet absorbance at 570 nm. (**F**) Representative transwell assay images showing the migration of METTL3- and METTL14-depleted cells. Scale bars, 200 μm. (**G** and **H**) Quantification of migrated cells from (F) via ImageJ. (**I** and **J**) Proportion of live (annexin V−) and apoptotic (annexin V+) cells in METTL3-depleted (I) and METTL14-depleted (J) cells relative to Scr. (**K** and **L**) Cell cycle distribution of cells following METTL3 (K) and METTL14 knockdown (L). (**M**) Kaplan-Meier curve representing the tumor-free survival of mice subcutaneously transplanted with Scr, METTL3-depleted, or METTL14-depleted OVCAR8 cells. (**N**) Quantification of the bioluminescence signal over the tumor region at day 0 and day 48, indicating tumor progression per group. (**O**) Representative images of collected tumors at the end of the experiment. Scale bar, 1 cm. (**P** and **Q**) Scatterplots representing *METTL3* (P) and *METTL14* (Q) mRNA levels in normal and ovarian cancer tissues (GEO dataset GSE37582). (**R** and **S**) Kaplan-Meier curves for the overall survival for METTL3 (R) and METTL14 (S) expression in HGSOC using TCGA data. Xenograft experiments included 10 tumors per experimental group. Statistical analysis: two-tailed Student’s *t* test [(A), (B), (D), (E), (G), (H), (I), (J), (K), (L), (P), and (Q)] and one-way ANOVA with Dunnett’s correction for multiple comparisons (N). Data are mean ± SD; *n* = 3 [(A), (B), (D), (E), (G), (H), (I), (J), (K), (L), (P), and (Q)], independent biological experiments. n.s., not significant.

To assess the tumorigenic roles of METTL3 and METTL14 in vivo, luciferase-labeled OVCAR8 cells, with either knockdown of METTL3, METTL14, or a Scr control, were subcutaneously injected into nude mice. Mice injected with control OVCAR8 cells rapidly developed tumors, with a median tumor-free survival of 12 days. In contrast, mice injected with METTL3- or METTL14-depleted cells exhibited notably delayed tumor onset, with tumor-free survival extending to 21 and 28 days, respectively (Fig. 1M). Bioluminescence imaging further demonstrated that tumors from METTL3- and METTL14-deficient cells grew at a markedly reduced rate compared to control tumors (Fig. 1N and fig. S2I). After 8 weeks, excised tumors showed substantially lower volume and weight in the knockdown groups than in the controls (Fig. 1O and fig. S2, J and K), highlighting the critical role of METTL3 and METTL14 in promoting tumor growth and progression in ovarian cancer in vivo.

To assess METTL3 and METTL14 expression in ovarian cancer, we analyzed mRNA levels in 74 ovarian carcinomas and 47 normal ovarian samples from the GSE37582 dataset of the Gene Expression Omnibus (GEO). *METTL3* was notably up-regulated in ovarian cancer tissues compared to normal tissues, whereas *METTL14* did not show a marked change (Fig. 1, P and Q). Kaplan-Meier survival analyses using data from The Cancer Genome Atlas (TCGA) showed that high *METTL3* expression correlated with reduced overall survival across all ovarian cancer subtypes (fig. S2L). Although elevated METTL14 expression was associated with better outcomes, this trend was not statistically significant (fig. S2M). Specifically, in HGSOC, we found that high *METTL3* levels predicted poorer survival (Fig. 1R), whereas high *METTL14* expression showed a statistically nonsignificant trend toward worse prognosis (Fig. 1S). Collectively, these findings underscore the critical roles of METTL3 and METTL14 in ovarian cancer cell growth and migration. The impact of STM2457 highlights the therapeutic potential of targeting METTL3, which is supported by its up-regulation and association with poor prognosis. In contrast, despite its functional importance, METTL14 lacks clear prognostic relevance.

### METTL3- and METTL14-driven transcriptomic rewiring and global m^6^A mapping

To explore the transcriptional changes associated with METTL3 and METTL14 depletion, we performed RNA sequencing (RNA-seq) on OVCAR8 cells transduced with control or shRNAs targeting METTL3 or METTL14. Following METTL3 silencing, we identified 3448 and 4896 differentially expressed genes (DEGs) in sh1 and sh2, respectively (Fig. 2A; fig. S3, A and B; and data S1). Across both shRNAs, 743 were consistently up-regulated and 705 down-regulated (fig. S3, C and D). METTL14 depletion produced an even broader transcriptional shift, yielding 6117 and 5146 DEGs for sh1 and sh2, respectively (Fig. 2B; fig. S3, E and F; and data S2), with 905 up-regulated and 994 down-regulated genes shared between the two knockdowns (fig. S3, G and H). Gene Ontology (GO) analysis of the up-regulated genes revealed enrichment in a broad range of biological processes, including regulation of transcription by RNA polymerase II, chromatin remodeling, protein transport, ubiquitination, and in utero embryonic development (Fig. 2C). Conversely, down-regulated genes were more selectively enriched in cancer-associated pathways, such as signal transduction, regulation of cell proliferation, extracellular matrix organization, and negative regulation of cell migration (Fig. 2D).

**Fig. 2.**
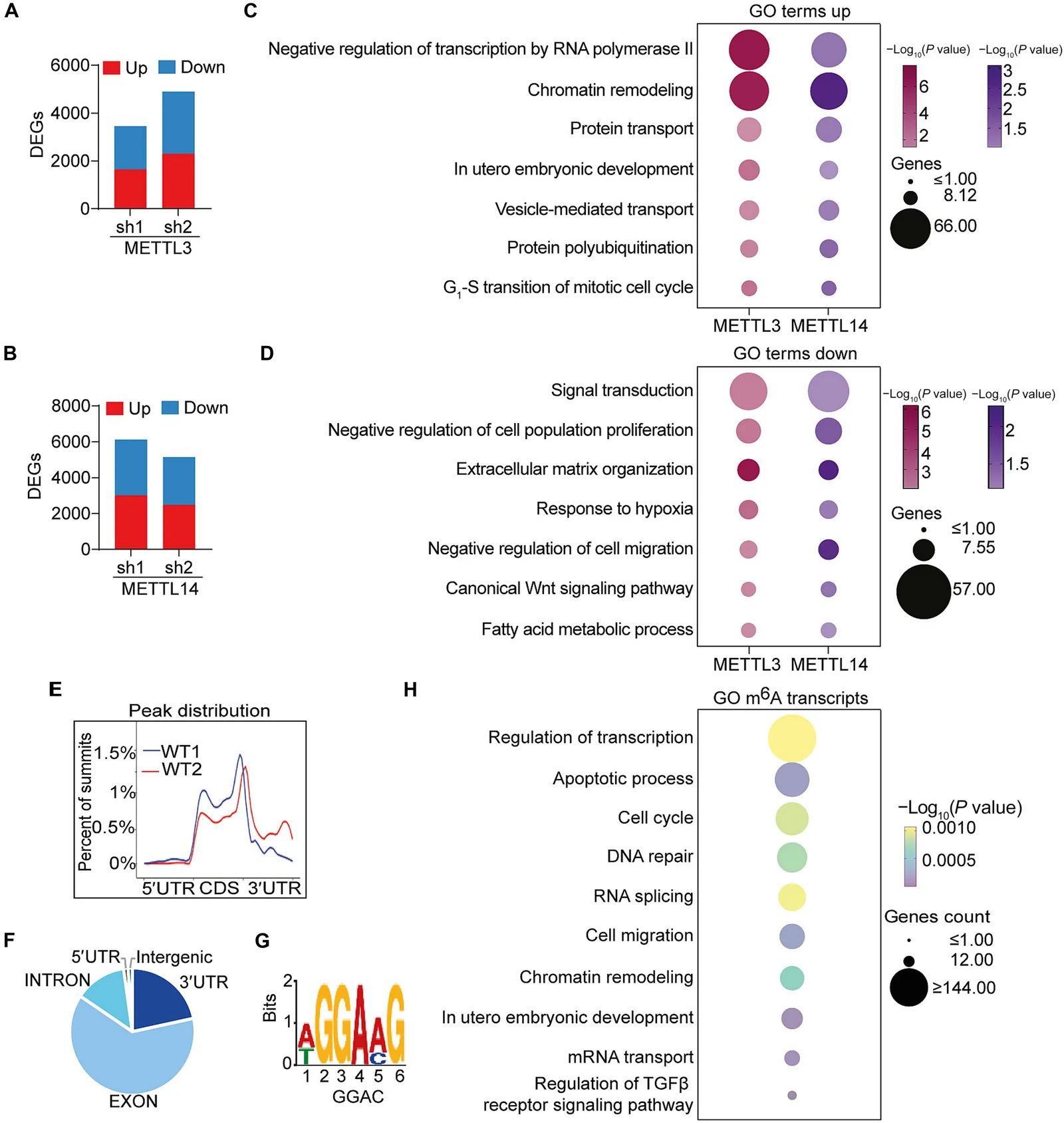
Transcriptomic and epitranscriptomic profiling reveals m^6^A-regulated gene expression programs in OVCAR8 cells. (**A** and **B**) Bar graphs show the number of DEGs identified with shRNAs targeting METTL3 (A) and METTL14 (B). (**C** and **D**) GO biological process enrichment analysis of shared up-regulated (C) and down-regulated (D) genes after METTL3 and METTL14 depletion, visualized by the dot plot. (**E**) Density distribution of m^6^A peaks across the mRNA transcripts, including 5′UTR, CDS, and 3′UTR. (**F**) Pie chart illustrating the distribution of m^6^A peaks across genomic regions, including introns, 5′UTR, intergenic, 3′UTR, and exon. (**G**) m^6^A motif detected by the MEME motif analysis with MeRIP-seq data in OVCAR8 cells. (**H**) GO dot plot showing enriched biological functions of m^6^A-modified transcripts identified in MeRIP-seq data. Data are from *n* = 2 independent biological experiments.

To determine whether these transcriptional changes are linked to m^6^A RNA methylation, we performed m^6^A RNA immunoprecipitation followed by sequencing (MeRIP-seq) in OVCAR8 cells to map m^6^A-modified transcripts in HGSOC cells. A total of 6178 m^6^A peaks were identified, primarily enriched near stop codons of protein-coding transcripts and within the coding sequence (CDS) (Fig. 2, E and F, and data S3). Sequence analysis confirmed the presence of the RRACH consensus motif (R = G or A; H = A, C, or U; A could be converted to m^6^A) (Fig. 2G). Integration of the RNA-seq and MeRIP-seq datasets showed that most m^6^A-modified transcripts were up-regulated upon METTL3 silencing (fig. S3, I and J). Although this is an indirect measure, it suggests increased transcript stability, consistent with one of the primary roles of m^6^A in regulating RNA decay, among its diverse functions in RNA metabolism. GO analysis of m^6^A-modified transcripts was enriched in pathways critical to cancer, including transcriptional regulation, apoptotic processes, cell cycle control, DNA repair, cell migration, in utero embryonic development, mRNA transport, and regulation of transforming growth factor–β (TGFβ) receptor signaling (Fig. 2H), supporting a role of m^6^A modification in modulating gene expression programs in ovarian cancer.

### m^6^A promotes DNMT1 translation

MeRIP-seq analysis identified *DNMT1*, the maintenance DNA methyltransferase, as an m^6^A-modified transcript, with the modification site in exon 8 within its CDS (Fig. 3A). Given the central role of DNA methylation in gene expression, chromatin stability, and genomic imprinting, and its potential implication in early neoplastic transformation, we investigated the regulation of DNMT1 by m^6^A in ovarian cancer (*25*). m^6^A RNA immunoprecipitation followed by qPCR showed an ∼2.5-fold enrichment of m^6^A-modified *DNMT1* mRNA (Fig. 3B). For nucleotide-level resolution analysis, the m^6^A site within exon 8 at position 3013 was assessed using SELECT, a robust method for detecting locus-specific m^6^A modifications (*26*). STM2457-treated OVCAR8 cells showed a threefold increase in SELECT products, indicative of reduced m^6^A modification at this site (Fig. 3C). Comparable results were obtained upon METTL3 or METTL14 depletion (Fig. 3D and fig. S4A). To investigate the fate of this modification, we first examined the transcript stability. Following actinomycin D treatment, *DNMT1* mRNA levels remained unchanged over 48 hours in both STM2457-treated and METTL3-depleted cells relative to controls (fig. S4, B and C), indicating that m^6^A does not affect *DNMT1* transcript stability. In contrast, DNMT1 protein levels were notably reduced upon METTL3 or METTL14 depletion and following STM2457 treatment, without corresponding changes in mRNA levels (Fig. 3, E to H, and fig. S4, D and E), supporting a role in translational control. Notably, METTL14 knockdown with shRNA_1 increased *DNMT1* mRNA levels (fig. S4D), suggesting a compensatory feedback mechanism. DNMT1 protein was also reduced in METTL3- and METTL14-depleted mouse tumor tissues (Fig. 3, I and J). Moreover, a statistically significant positive correlation between METTL3 and DNMT1 expression was also observed in ovarian cancer tissues but not in normal tissues, supporting a cancer-specific m^6^A-dependent regulation of DNMT1 (Fig. 3, K to M).

**Fig. 3.**
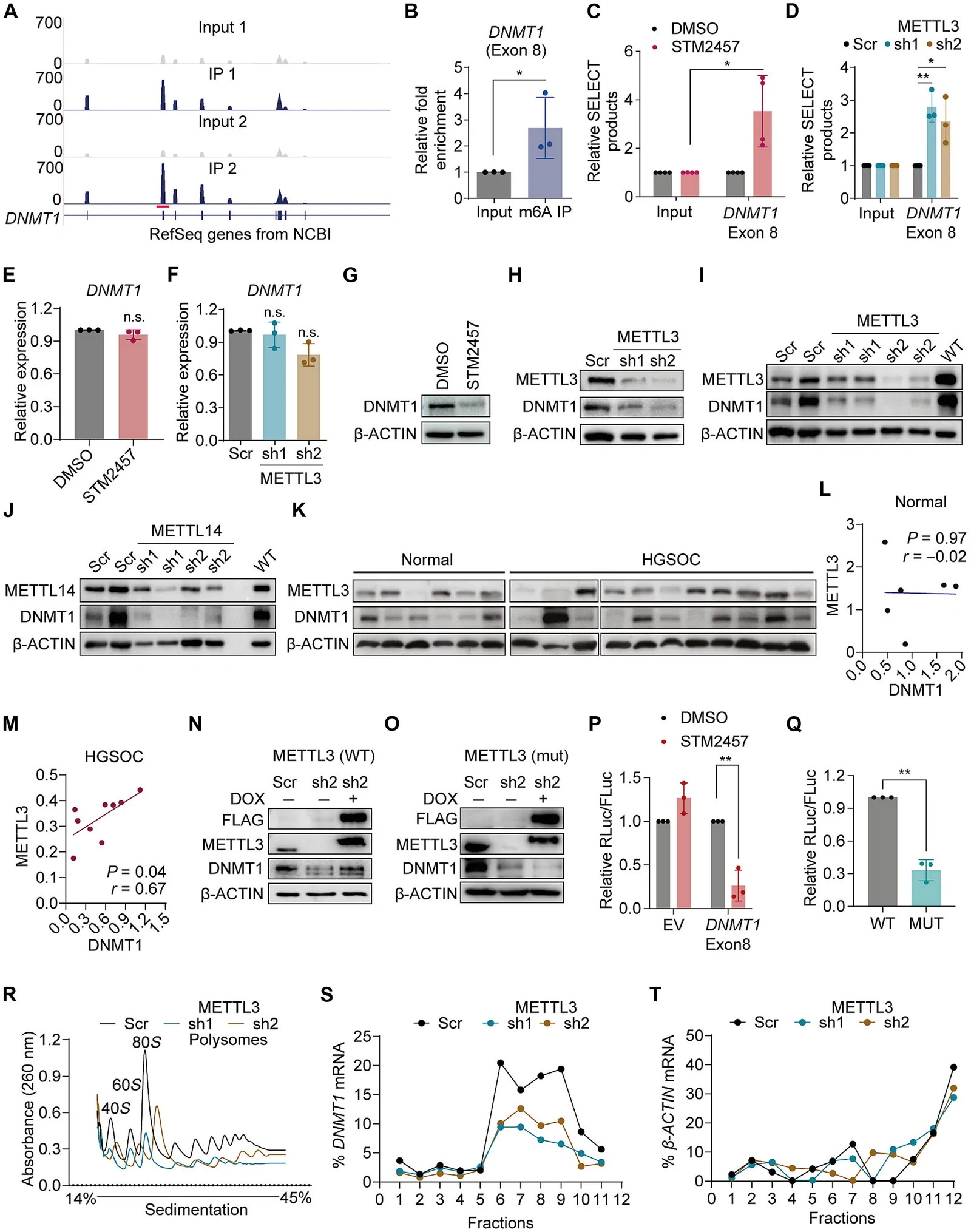
METTL3-mediated m^6^A modification promotes DNMT1 translation. (**A**) IGV track showing m^6^A peak distribution across DNMT1 mRNA exon 8, comparing m^6^A-immunoprecipitated RNA (blue) to input control (gray). (**B**) Bar graph of DNMT1 fold enrichment following m^6^A IP versus input control. (**C** and **D**) Bar graphs of SELECT product levels following STM2457 treatment (C) and METTL3 depletion (D). (**E** and **F**) Bar graphs of DNMT1 mRNA expression following STM2457 treatment (E) and METTL3 depletion (F) by qRT-PCR. (**G** and **H**) Western blots of DNMT1 protein levels in STM2457-treated (G) and METTL3-depleted (H) cells. (**I** and **J**) Western blots of DNMT1 expression in xenografts protein extracts from METTL3- (I) and METTL14-depleted (J) cells. (**K**) METTL3 and DNMT1 expression in normal ovarian epithelial and HGSOC tissues. (**L** and **M**) Correlation plots of DNMT1 and METTL3 protein levels in normal ovarian (L) and HGSOC tissues (M), quantified from (K). (**N** and **O**) Western blots of FLAG, METTL3, and DNMT1 protein levels in Scr, sh2METTL3, and sh2METTL3 cells expressing doxycycline (DOX)–inducible FLAG-tagged WT METTL3 (N) and catalytic mutant METTL3 (O). (**P**) Relative Renilla luciferase activity (normalized to FLuc) in cells transfected with an empty vector (EV) or psiCHECK2-DNMT1-Exon 8 and treated with DMSO or STM2457 for 48 hours. (**Q**) RLuc/FLuc activity in cells transfected with psiCHECK2-DNMT1-Exon 8 WT versus m^6^A-mutant (A-to-C; MUT) constructs. (**R**) Polysome profiling curve showing global ribosomal distribution in METTL3-depleted cells. (**S** and **T**) qRT-PCR of *DNMT1* (S) and β-*actin* (T) mRNA across polysome fractions. β-ACTIN served as the Western blot loading control. Statistical analysis: two-tailed Student’s *t* test [(B), (C), (D), (E), (F), and (Q)], one-way ANOVA with Dunnett’s correction for multiple comparisons (P), and Pearson’s correlation coefficient (*r*) analysis [(L) and (M)]. Data are mean ± SD; *n* = 3 [(B), (C), (D), (E), (F), (P), and (Q)] independent biological experiments.

We next examined the specificity of our findings using overexpression and rescue approaches. Overexpression of wild-type (WT) METTL3 increased DNMT1 protein levels, whereas a catalytically inactive METTL3 mutant reduced them (fig. S4F). In OVCAR8 cells expressing a FLAG-tagged, shRNA-resistant, doxycycline-inducible METTL3 construct, DNMT1 protein levels were restored following METTL3 knockdown (Fig. 3N). In contrast, expression of a catalytically inactive METTL3 further decreased DNMT1 levels beyond those observed with METTL3 knockdown alone (Fig. 3O), confirming that the METTL3 methyltransferase activity is required to sustain DNMT1 protein expression. Consistently, METTL14 overexpression also increased DNMT1 protein levels (fig. S4G).

To further investigate whether m^6^A modification regulates DNMT1 translation, we transfected OVCAR8 cells with a dual-luciferase reporter containing the *DNMT1* Exon 8 downstream of Renilla luciferase. STM2457 treatment considerably reduced Renilla activity, suggesting that m^6^A modification within *DNMT1* Exon 8 is sufficient to influence its translational output (Fig. 3P). Mutation of the identified m^6^A site decreased baseline luciferase activity (Fig. 3Q) and attenuated responsiveness to STM2457 compared to the WT construct (fig. S4H), demonstrating that this site is required for METTL3-dependent translation enhancement. Polysome profiling of METTL3-depleted cells led to a global reduction in ribosomal subunits, monosomes, and polysomes compared to the control cells (Fig. 3R and fig. S4, I and J), suggesting an overall impairment in translation. To assess whether this reflects impaired ribosome biogenesis, we examined ribosomal RNA (rRNA) levels and processing. Agarose gel analysis revealed reduced levels of mature 28*S* and 18*S* rRNAs upon METTL3 depletion (fig. S4K). Northern blot analysis of pre-rRNA processing intermediates further showed a marked reduction in both early and late precursors of the 18*S* pathway, including 30*S* pre-rRNA and the 26*S* and 21*S* ITS1-derived intermediates (fig. S4L). Similarly, reduced levels of 32*S* precursors were observed in the 28*S*/5.8*S* pathway (fig. S4M), indicating defective ribosome biogenesis and reduced ribosome abundance. qPCR analysis of polysome fractions further revealed a decrease association of *DNMT1* mRNA with polysomes (Fig. 3S and fig. S4, N and O), whereas β-*ACTIN* mRNA, serving as a control, remained unaffected (Fig. 3T and fig. S4, P and Q). Although reduced DNMT1 polysome association may partly result from decreased ribosome availability, luciferase assays support a direct m^6^A-dependent mechanism as DNMT1 Exon 8–driven translation was reduced by STM2457 (Fig. 3P) and attenuated upon mutation of the m^6^A motif (Fig. 3Q and fig. S4H), under conditions not confounded by global ribosome depletion.

To examine METTL3-DNMT1 interactions at the chromatin level in HGSOC, and to contextualize our findings relative to the recently described METTL3-METTL14-DNMT1 chromatin axis (*27*), we confirmed by coimmunoprecipitation a physical interaction between endogenous METTL3 and DNMT1 in OVCAR8 cells (fig. S4R). METTL3 knockdown reduced DNMT1 chromatin occupancy, consistent with both decreased total DNMT1 protein levels and impaired chromatin recruitment (fig. S4S). Pharmacologic inhibition of METTL3 produced a more modest reduction in chromatin-associated DNMT1 (fig. S4T), likely reflecting a partial decrease in total DNMT1 protein levels rather than a direct disruption of chromatin recruitment. Collectively, these findings support a model in which m^6^A modification enhances DNMT1 translation, highlighting a key posttranscriptional mechanism by which METTL3 regulates DNMT1 protein levels while not excluding the possibility that additional mechanisms, including regulation of chromatin recruitment, also contribute.

### DNMT1 promotes ovarian cancer growth

Having established that METTL3 enhances DNMT1 translation, we next investigated the functional relevance of DNMT1 in ovarian cancer. Analysis of the GEO dataset GSE37582 revealed no notable difference in *DNMT1* mRNA levels between ovarian cancer and normal tissues (Fig. 4A). Likewise, analysis of TCGA datasets showed no notable association between *DNMT1* expression and overall survival across all ovarian cancer subtypes (fig. S5A). Although not statistically significant, increased *DNMT1* expression showed a trend toward poorer survival specifically in HGSOC (fig. S5B), indicating that transcripts levels may not fully capture the biological and clinical significance of DNMT1 in this subtype. Therefore, to evaluate DNMT1 at the protein level, we performed immunohistochemical (IHC) analysis of tissue microarrays (TMAs) comprising 180 ovarian carcinoma specimens representing multiple histological subtypes (Fig. 4B). In contrast to the unchanged mRNA levels (Fig. 4A), DNMT1 protein expression was considerably higher in HGSOC than in low-grade serous carcinoma (LGSC), supporting a pathogenic role for DNMT1 in this aggressive subtype (Fig. 4C). To assess the consequences of DNMT1 inhibition, we treated OVCAR8 cells with GSK3484862, a selective DNMT1 inhibitor that promotes proteasome-dependent DNMT1 degradation without affecting its mRNA levels (Fig. 4, D and E) (*28*). Dot blot confirmed effective suppression of DNMT1 activity, as evidenced by a marked reduction in 5mC levels (Fig. 4F). Phenotypically, GSK3484862 treatment reduced cell growth and migration, induced apoptosis, and caused S-phase cell cycle arrest (Fig. 4, G to K).

**Fig. 4.**
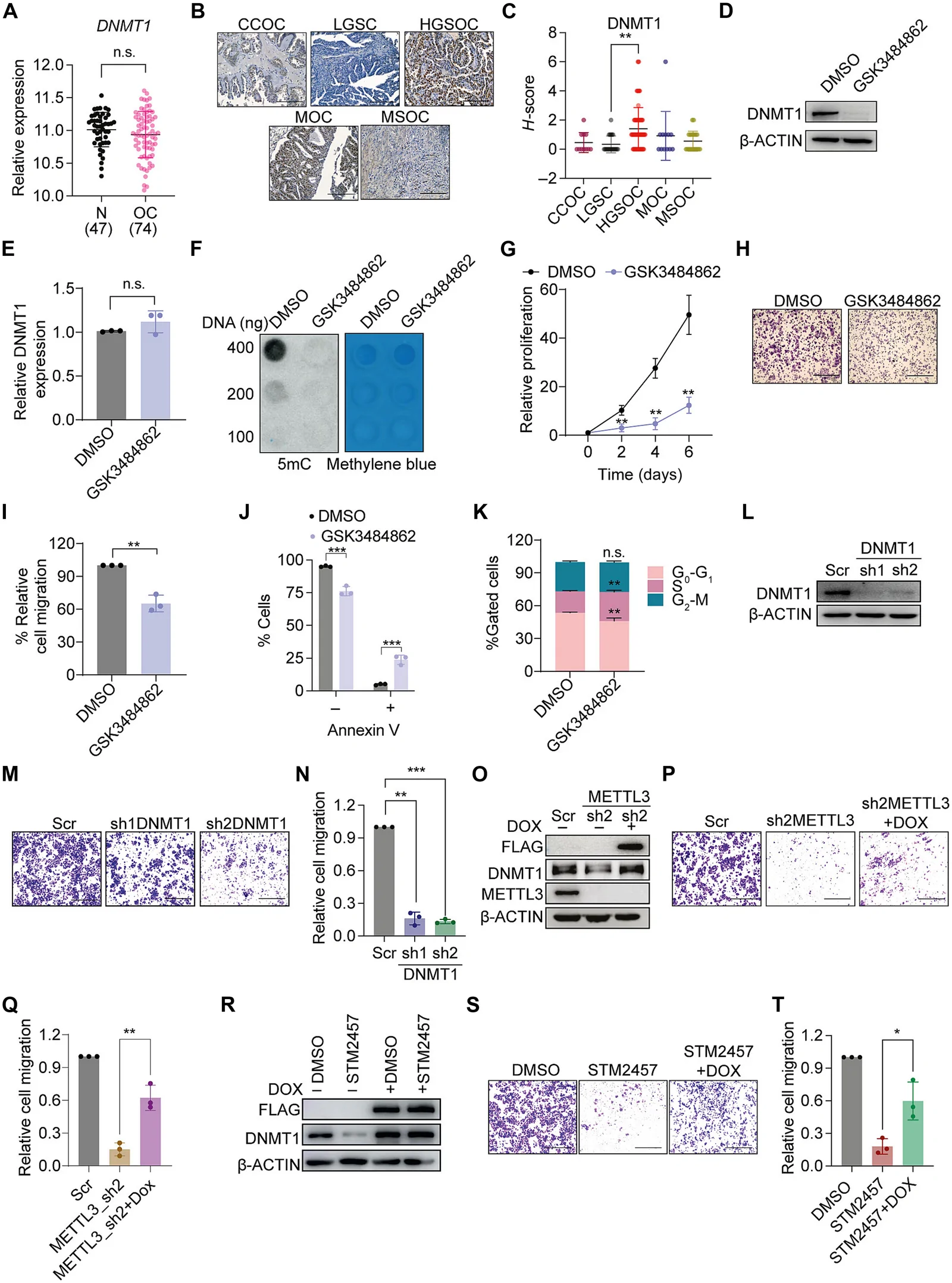
DNMT1 is up-regulated in HGSOC and promotes tumor cell migration. (**A**) *DNMT1* expression levels in GSE37582. (**B**) Representative IHC images of DNMT1 expression across ovarian cancer subtypes. CCOC, clear cell ovarian carcinoma; LGSC, low-grade serous carcinoma; HGSOC, high-grade serous carcinoma; MOC, mucinous ovarian carcinoma; MSOC, metastatic ovarian carcinoma. Scale bars, 50 μm. (**C**) DNMT1 IHC *H* score in patient tissues from (B). (**D** and **E**) Immunoblot (D) and qRT-PCR (E) of DNMT1 expression in GSK3484862-treated cells. (**F**) Dot blot of 5mC levels in DMSO- and GSK3484862-treated cells; methylene blue as a DNA loading control. (**G**) Cell proliferation in DMSO- and GSK3484862-treated cells over 6 days. (**H**) Transwell images of migration in DMSO and GSK3484862-treated cells. Scale bars, 200 μm. (**I**) Quantification of migrated cells from (H), by crystal violet absorbance at 570 nm. (**J**) Proportion of live (annexin V−) and apoptotic (annexin V+) cells in DMSO- and GSK3484862-treated cells. (**K**) Cell cycle distribution following GSK3484862 treatment. (**L**) Immunoblot of DNMT1 shRNAs knockdown efficiency. (**M**) Transwell images of DNMT1-depleted cell migration. Scale bars, 200 μm. (**N**) Relative cell migration quantified from (M). (**O**) Immunoblots of FLAG, DNMT1, and METTL3 levels in Scr, sh2METTL3, and sh2METTL3 cells expressing DOX-inducible FLAG-tagged DNMT1. (**P** and **Q**) Images (P) and graph (Q) of cell migration in Scr control, sh2METTL3, and sh2METTL3+DOX-inducible FLAG-DNMT1 cells. (**R**) Immunoblots of FLAG and DNMT1 levels in DMSO- and STM2457-treated, with and without DOX-inducible FLAG-tagged DNMT1 expression. (**S** and **T**) Images (S) and graph (T) of cell migration in DMSO, STM2457-treated, and STM2457+DOX-inducible FLAG-DNMT1 cells. Scale bars, 200 μm. Statistical analysis: two-tailed Student’s *t* test [(A), (E), (G), (I), (J), (N), (Q), and (T)] and one-way ANOVA with Dunnett’s correction for multiple comparisons (C). Data are mean ± SD; *n* = 3 [(D) to (T)] independent biological experiments.

To exclude potential off-target effects of pharmacological inhibition and validate these findings using an orthogonal genetic approach, we performed shRNA-mediated DNMT1 knockdown in OVCAR8 cells (Fig. 4L). In contrast to GSK3484862 treatment, DNMT1 knockdown did not impair proliferation or induce apoptosis and instead showed a trend toward increased proliferation (fig. S5, C and D). However, consistent with the GSK3484862 treatment, DNMT1 depletion substantially reduced cell migration (Fig. 4, M and N). These findings suggest that loss of DNMT1 is sufficient to suppress migration but does not fully account for the antiproliferative and proapoptotic effects observed following acute pharmacological inhibition. In line with this, rescue experiments using a doxycycline-inducible DNMT1 construct in cells subjected to either METTL3 knockdown or STM2457 treatment demonstrated that DNMT1 reexpression failed to rescue the reduced proliferation or enhanced apoptosis caused by METTL3 loss (fig. S5, E and H) but considerably restored migration (Fig. 4, O to T). These observations are consistent with the phenotype observed following DNMT1 knockdown and indicate that DNMT1 is a major mediator of METTL3-dependent cell migration. In contrast, the effects of METTL3 on proliferation and apoptosis likely involve additional downstream targets. Collectively, these results identify DNMT1 as an important, but not exclusive, effector of METTL3-driven oncogenic phenotypes in HGSOC, reflecting the broad regulatory impact of m^6^A modification across the transcriptome.

### The METTL3-DNMT1 axis silences tumor suppressor genes in ovarian cancer

To identify DNMT1 target genes in HGSOC, we conducted RNA-seq on OVCAR8 cells following treatment with the DNMT1 inhibitor GSK3484862. This analysis identified 222 DEGs, including 196 up-regulated and 26 down-regulated transcripts (Fig. 5A and data S4). The predominance of up-regulated genes is consistent with DNMT1 functioning primarily as a transcriptional repressor, as previously reported in other cellular contexts (*29*, *30*), and supports a similar role in HGSOC. GO analysis of the up-regulated genes showed enrichment in biological processes such as transcriptional regulation, apoptosis, chromatin remodeling, negative regulation of cell population proliferation, and response to hypoxia, among others (Fig. 5B). To determine whether the transcriptional derepression was associated with changes in DNA methylation, we performed methylation array profiling on GSK3484862-treated OVCAR8 cells. t-distributed stochastic neighbor embedding (t-SNE) analysis showed a clear separation between dimethyl sulfoxide (DMSO)– and GSK3484862-treated samples (fig. S6A). Methylation density plots displayed a bimodal distribution, with a higher peak for unmethylated CpGs in treated samples, reflecting a genome-wide loss of DNA methylation following DNMT1 inhibition (fig. S6B). In line with this, mean methylation levels were substantially lower in GSK3484862-treated cells than in the controls (fig. S6C). Unsupervised clustering further confirmed widespread hypomethylation following DNMT1 inhibition (fig. S6D). Consistently, 11,141 hypomethylated CpG sites were identified in response to GSK3484862 treatment (Fig. 5C).

**Fig. 5.**
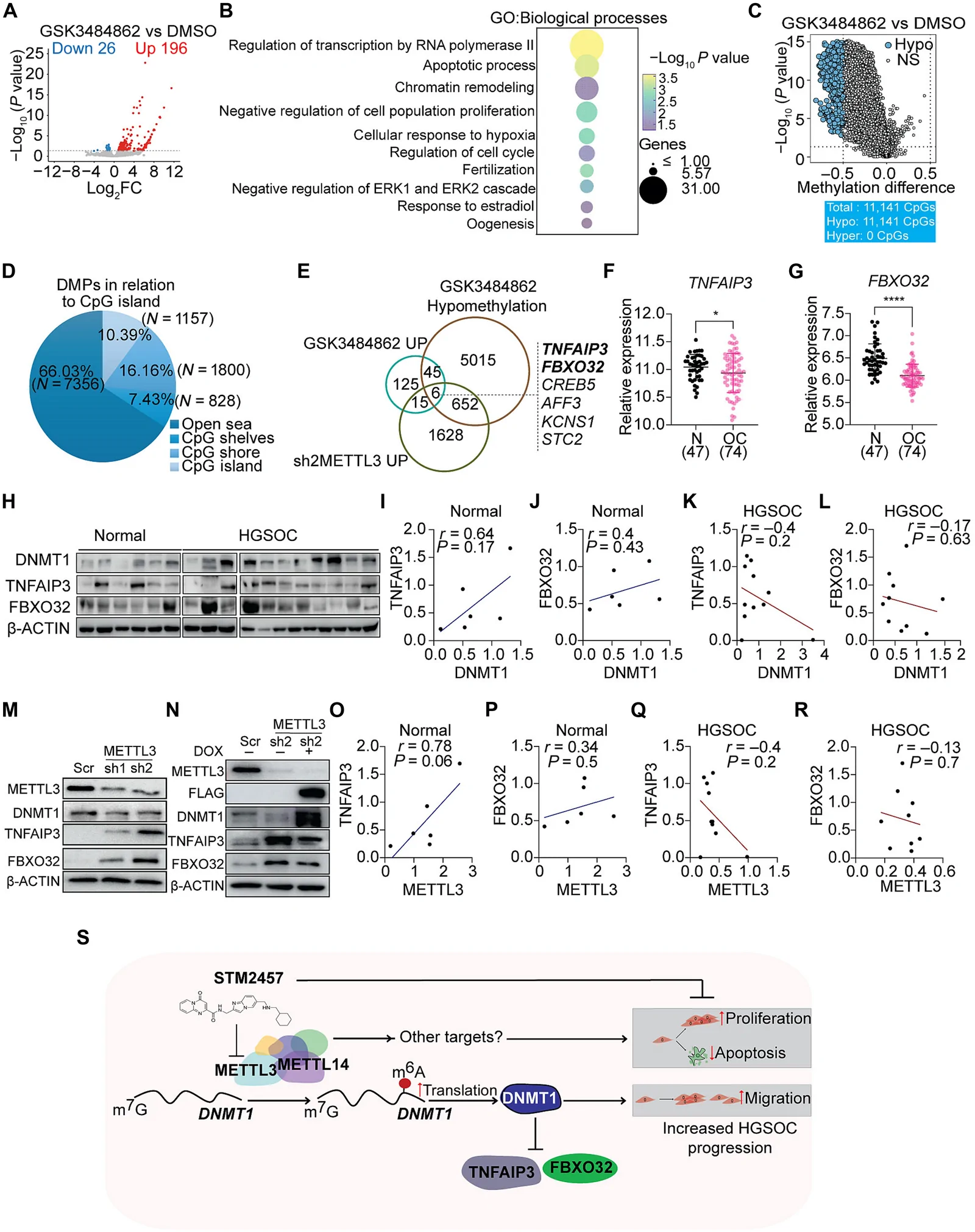
The METTL3-DNMT1 axis silences tumor suppressor genes TNFAIP3 and FBXO32 in ovarian cancer. (**A**) Volcano plot of DEGs in DMSO- and GSK3484862-treated cells, highlighting up-regulated (red) and down-regulated (blue) genes. FC, fold change. (**B**) Dot plot of enriched GO terms for genes up-regulated upon GSK3484862 treatment. (**C**) Plot of methylation difference in DMSO- and GSK3484862-treated cells. Differentially methylated CpG (DMPs) defined as adjusted *P* value ≤ 0.05 and mean methylation difference ≥ 0.5 between conditions. (**D**) Pie chart of differentially methylated probe distribution relative to CpG island location. (**E**) Venn diagram of the overlap between up-regulated genes, hypomethylated CpG-associated genes following GSK3484862 treatment, and genes up-regulated following METTL3 depletion (sh2). (**F** and **G**) Scatterplots of *TNFAIP3* (F) and *FBXO32* (G) expression in ovarian cancer versus normal tissue (GSE37582). (**H**) Immunoblots of DNMT1, TNFAIP3, and FBXO32 in normal ovarian epithelial and ovarian cancer tissues. (**I** to **L**) Correlation plots of DNMT1 and TNFAIP3 [(I) and (K)] and FBXO32 [(J) and (L)] in normal [(I) and (J)] and ovarian cancer tissues [(K) and (L)], quantified from (H) by ImageJ. (**M**) Immunoblots of METTL3, DNMT1, TNFAIP3, and FBXO32 in METTL3-depleted cells. (**N**) Western blots of METTL3, FLAG, DNMT1, TNFAIP3, and FBXO32 in Scr, sh2METTL3, and sh2METTL3+DOX-inducible FLAG-DNMT1-expressing cells. (**O** to **R**) Correlation plots of METTL3 (Fig. 3K) and TNFAIP3 [(O) and (Q)] and FBXO32 [(P) to (R)] in normal [(O) and (P)] and ovarian cancer tissues [(Q) and (R)]. (**S**) Schematic of the METTL3-DNMT1 axis in ovarian tumor progression. For Western blot analysis, β-ACTIN served as the Western blot loading control. Statistical analysis: two-tailed Student’s *t* test [(F) and (G)] and Pearson’s correlation coefficient (*r*) analysis [(I) to (L) and (O) to (R)]. Data are mean ± SD; *n* = 3 [(A) to (E), (M), and (N)] independent biological experiments.

In contrast, STM2457-treated samples showed no discernible differences compared to DMSO-treated controls. t-SNE analysis revealed substantial overlap between these conditions, with one STM2457 and one DMSO sample intermixing, indicating minimal effects on global DNA methylation (fig. S6, A to D). This suggests that residual DNMT1 levels following METTL3 inhibition remain sufficient to maintain 5mC levels (*31*). Most hypomethylated CpGs identified after DNMT1 inhibition were found in open sea regions (66.03%), whereas smaller fractions were associated with CpG islands (10.39%), shelves (7.43%; 2 to 4 kb from the promoter CpG islands), and shores (16.16%; 0 to 2 kb from the promoter CpG islands) (Fig. 5D). In terms of gene location, 49.65% of differentially methylated CpG sites were found within gene bodies, whereas 25.2% in 5′ regulatory regions, 21.65% in intergenic regions, and 3.49% in 3′ untranslated regions (3′UTRs) (fig. S6E). When analyzed by gene type, most were located in coding genes (72.14%), with fewer in intergenic (21.65%) and noncoding genes (6.21%) (fig. S6F).

To identify genes whose transcriptional reactivation upon DNMT1 inhibition was directly linked to loss of DNA methylation, we integrated RNA-seq and methylation array datasets. This analysis identified 51 (26%) overlapping transcripts that were both up-regulated and hypomethylated upon DNMT1 inhibition (Fig. 5E). The limited overlap between widespread genome-wide hypomethylation and transcriptional reactivation is consistent with the well-established observation that changes in DNA methylation and gene expression are often discordant at the genome scale, with only a subset of methylation alterations having direct transcriptional effects (*32*–*34*). This finding suggests that DNA methylation loss alone is insufficient to reactivate most silenced loci, pointing to the existence of additional reinforcing repressive mechanisms. To further investigate whether these transcripts were also regulated by METTL3, we focused on the more efficient knockdown achieved by sh2 METTL3 (Fig. 5E). Among the overlapping genes, we prioritized tumor necrosis factor alpha-induced protein 3 (TNFAIP3) and F-Box Protein 32 (FBXO32) because of their well-characterized tumor suppressor roles (*35*–*41*). Of note, these transcripts are not m^6^A modified, indicating that they are not direct targets of METTL3 but rather downstream effectors of the METTL3-DNMT1 regulatory axis. To explore their relevance in ovarian cancer, we first analyzed TNFAIP3 and FBXO32 expression using the GEO dataset GSE37582. Both genes exhibited consistent down-regulation in ovarian cancer tissues compared to controls, consistent with their tumor suppressor roles (Fig. 5, F and G). Survival analysis using TCGA data revealed a trend toward improved survival in patients with high TNFAIP3 and FBXO32 expression, particularly in the HGSOC subtype, although this did not reach statistical significance (fig. S7, A to D). Analysis of patient-derived ovarian tumor specimens further showed elevated DNMT1 expression, concomitant with reduced TNFAIP3 and FBXO32 expression, compared with normal tissues (Fig. 5H). Although not statistically significant, in normal ovarian samples, DNMT1 expression showed a positive correlation with TNFAIP3 and FBXO32 (Fig. 5, I and J). Conversely, this relationship was reversed in ovarian cancer, where DNMT1 expression negatively correlated with the expression of both genes (Fig. 5, K and L). These findings suggest that DNMT1-mediated epigenetic regulation contributes to the transcriptional repression of TNFAIP3 and FBXO32 in ovarian cancer.

Given this inverse correlation, we next sought to evaluate whether METTL3 modulates TNFAIP3 and FBXO32 expression. METTL3 knockdown increased the protein levels of both TNFAIP3 and FBXO32, indicating negative regulation by METTL3 (Fig. 5M). Similarly, inhibition of METTL3 with STM2457 elevated the expression of both genes (fig. S7E). Notably, DNMT1 overexpression in METTL3 knockdown cells reduced TNFAIP3 and FBXO32 protein levels compared to knockdown alone, further supporting that METTL3 acts upstream of DNMT1 to repress these tumor suppressor genes (Fig. 5N). Moreover, correlation analysis revealed a positive association between METTL3 and these tumor suppressor genes in normal ovarian tissue, which shifted to a negative correlation in ovarian cancer samples (Fig. 5, O to R), although these associations were not statistically significant. Together, these trends suggest a coordinated regulatory mechanism involving the METTL3-DNMT1 axis.

To determine whether this regulatory relationship extends beyond OVCAR8 cells, we depleted METTL3 or DNMT1 in a panel of cell lines derived from distinct tissue types, including OVCAR4 (ovarian cancer), MDA-MB-231 (breast cancer), HEK293T (embryonic kidney), SUIT2 and MiaPaCa2 (pancreatic cancer), and U2OS (osteosarcoma). Across all cell lines examined, METTL3 depletion reduced DNMT1 expression and was accompanied by increased TNFAIP3 and FBXO32 expression (fig. S7F). Similarly, shRNA-mediated DNMT1 depletion increased TNFAIP3 and FBXO32 expression across multiple cellular contexts (fig. S7G). These findings indicate that regulation of the DNMT1-TNFAIP3/FBXO32 axis by METTL3 is not restricted to ovarian cancer cells and may represent a more general regulatory mechanism.

To determine whether TNFAIP3 and FBXO32 functionally mediate the tumor-suppressive effects of METTL3 inhibition, we simultaneously silenced both genes using small interfering RNAs (siRNAs) in the context of either METTL3 inhibition (STM2457) or DNMT1 inhibition (GSK3484862) and assessed effects on cell proliferation. Knockdown efficiency was confirmed by Western blotting (fig. S7, H and I). Cosuppression of TNFAIP3 and FBXO32 notably rescued the reduction in cell proliferation induced by STM2457 treatment (fig. S7J), demonstrating that their up-regulation contributes to the antitumorigenic effects of METTL3 inhibition and establishing them as key downstream effectors of the METTL3-DNMT1 axis. Nonetheless, we do not exclude the possibility that additional genes or pathways downstream of METTL3 also contribute to this phenotype. In contrast, cosuppression of TNFAIP3 and FBXO32 failed to rescue the antiproliferative effects of DNMT1 inhibition (fig. S7K). This indicates that the antiproliferative effect of GSK3484862 is so potent that it cannot be overcome solely by silencing these two genes, consistent with its broader role as a global epigenetic repressor acting on a wide range of target genes. The comparatively stronger and nonrescuable suppression observed upon DNMT1 inhibition suggests that additional DNMT1 target genes are derepressed under these conditions and may further reinforce tumor-suppressive programs independently of TNFAIP3 and FBXO32. Collectively, these results identify TNFAIP3 and FBXO32 as key effectors of METTL3 inhibition while indicating that DNMT1 inhibition acts through a wider set of tumor-suppressive targets, together supporting a model in which METTL3 maintains DNMT1 activity to silence tumor suppressor programs in HGSOC (Fig. 5S).

## DISCUSSION

RNA modifications, particularly m^6^A, are now recognized as critical regulators of gene expression. In ovarian cancer, dysregulation of m^6^A “writers,” “readers,” and “erasers” has been implicated in tumorigenesis, metastasis, and chemoresistance (*13*–*19*, *42*–*53*). Our study builds on this model by revealing that METTL3 and METTL14 contribute to oncogenic programs in HGSOC, at least partially, through translational control of DNMT1. Depletion of either METTL3 or METTL14 impaired the proliferation and metastatic capacity of ovarian cancer cells both in vitro and in vivo, resulting in reduced tumor growth in xenograft models. Pharmacological inhibition of METTL3 using STM2457 recapitulated these effects in cells, highlighting the therapeutic potential of targeting the m^6^A machinery in HGSOC. METTL3 and METTL14 form the core m^6^A methyltransferase complex and are functionally interdependent for m^6^A deposition, complicating efforts to distinguish their individual contributions to tumor biology. Nevertheless, transcriptomic analyses following individual knockdown revealed partially distinct gene expression programs, consistent with emerging evidence for m^6^A-independent, moonlighting functions of these factors (*24*, *54*, *55*). Notably, analyses of TCGA datasets further showed that METTL3, but not METTL14, is transcriptionally up-regulated in ovarian cancer, pointing to differential modes of regulation in this disease context.

Consistent with a previous study (*43*), m^6^A profiling revealed enrichment of m^6^A sites within the CDS and near stop codons, and such modified transcripts were associated with pathways relevant to ovarian development and cancer progression. Among these, *DNMT1* emerged as a prominent target, harboring m^6^A sites within its CDS. Although increased DNMT1 expression has been previously correlated with m^6^A methylation in non—small cell lung cancer (*56*), direct evidence for m^6^A-mediated regulation was lacking. Here, we demonstrate that m^6^A enhances DNMT1 expression through translational control as METTL3 suppression reduced DNMT1 protein levels without affecting *DNMT1* mRNA abundance. This conclusion is supported by multiple complementary approaches showing reduced translational efficiency of DNMT1 following METTL3 suppression, in line with a recent report indicating that m^6^A modifications within the CDS can enhance translation in ovarian cancer (*43*), likely by resolving RNA secondary structures that impede elongation (*57*). However, CDS-localized m^6^A can also promote translation-coupled RNA decay under specific conditions (*58*–*60*), underscoring the context-dependent nature of m^6^A-mediated regulation and highlighting the need to evaluate its functional impact on a transcript-by-transcript basis.

Only a limited number of studies have addressed the cross-talk between m^6^A RNA modification and DNA methylation. Quarto *et al.* showed that the METTL3-METTL14 complex recruits DNMT1 to chromatin to mediate 5mC deposition in gene bodies (*27*), where gene body methylation is associated with transcriptional activation. Our study reveals a distinct but potentially complementary mechanism in HGSOC, whereby METTL3 and METTL14 regulate DNMT1 posttranscriptionally through m^6^A-dependent enhancement of translation, with DNMT1 functioning primarily as a transcriptional repressor through promoter silencing, as evidenced by the predominant gene up-regulation and promoter demethylation observed upon pharmacological DNMT1 inhibition. Nonetheless, we cannot exclude the possibility that the chromatin-associated roles of METTL3 and METTL14 also contribute to DNMT1 regulation in this context, particularly given our observation of METTL3-DNMT1 coimmunoprecipitation and reduced chromatin association upon silencing, although these effects may at least partly reflect overall reductions in DNMT1 protein levels. Other studies have shown that m^6^A readers regulate DNA methylation through TET enzyme recruitment: FXR1 recruits TET1 to promote local demethylation (*61*), and YTHDC2 similarly recruits TET1 to regulate transposable element activity (*62*). Thus, in previously described models, the relationship between m^6^A and DNA methylation operates through chromatin recruitment of DNMT1 or TET1. By contrast, the mechanism identified here operates primarily at the level of DNMT1 translational up-regulation. Furthermore, the downstream effects on DNA methylation differ across these models. Whereas disruption of the FXR1-TET1 axis decreases DNA demethylation and consequently favors accumulation of 5mC (*61*), loss of the METTL3-DNMT1 axis described by Quarto *et al.* reduces DNA methylation (*27*). These observations highlight the context-dependent nature of m^6^A-DNA methylation cross-talk. It is also worth noting that complete TET1 loss in mouse embryonic stem cells produces only a partial reduction in 5hmC (5-hydroxymethylcytosine) and a very subtle increase in global 5mC (*63*), suggesting that changes in m^6^A-dependent recruitment of DNA methylation machinery do not necessarily translate into large alterations in bulk DNA methylation.

In our hands, STM2457 treatment did not result in detectable changes in global 5mC levels despite reducing DNMT1 abundance. This observation is consistent with the threshold-dependent nature of DNMT1-mediated methylation maintenance, whereby partial reductions in DNMT1 levels are often insufficient to induce measurable genome-wide hypomethylation (*31*). In contrast, robust DNA hypomethylation was observed following GSK3484862 treatment, suggesting that more profound inhibition of DNMT1 activity is required before global methylation changes become apparent. The induction of TNFAIP3 and FBXO32 following METTL3 inhibition despite the absence of detectable DNA hypomethylation indicates that partial reductions in DNMT1 can still have considerable transcriptional consequences. Whether DNMT1 additionally contributes to gene silencing through noncatalytic scaffolding interactions with repressive chromatin complexes such as LSD1 and EZH2 warrants future investigation (*29*, *30*, *64*). One possibility is that the partial reduction in DNMT1 levels induced by METTL3 suppression is sufficient to derepress TNFAIP3 and FBXO32 through disruption of DNMT1-dependent repressive complexes, without necessarily producing detectable changes in global 5mC levels. Under this model, the widespread DNA hypomethylation observed following GSK3484862 treatment would represent a secondary layer of epigenetic disruption that only becomes apparent upon more profound inhibition of DNMT1 function. Although speculative, this framework may help explain why METTL3 depletion and DNMT1 inhibition share common transcriptional consequences despite differing effects on global DNA methylation.

Further illustrating the complexity of DNMT1 regulation, we recently showed that DNMT1 protein stability is controlled by LSD1 (KDM1A). Rather than acting through its canonical demethylase activity, LSD1 stabilizes DNMT1 through a scaffolding interaction with the deubiquitinase USP7, thereby protecting DNMT1 from proteasomal degradation (*65*). Together with the translational regulation described here, these findings indicate that DNMT1 abundance and function are controlled through coordinated posttranscriptional, posttranslational, and chromatin-associated mechanisms.

To directly establish the functional contribution of DNMT1 downstream of METTL3, we performed a series of orthogonal experiments. DNMT1 depletion recapitulated the up-regulation of TNFAIP3 and FBXO32 and impaired cell migration, whereas reexpression of DNMT1 restored repression of these genes and rescued migration in METTL3-depleted cells. In contrast, DNMT1 knockdown failed to reproduce, and DNMT1 reexpression failed to rescue the proliferation defects associated with METTL3 suppression. Together, these findings demonstrate that DNMT1 is a bona fide downstream effector of METTL3, mediating specific METTL3-dependent phenotypes including tumor suppressor gene repression and cell migration, whereas additional m^6^A-regulated targets likely contribute to the broader effects of METTL3 on proliferation and survival. Notably, regulation of the DNMT1-TNFAIP3/FBXO32 axis was conserved across multiple cancer cell lines, suggesting that this pathway extends beyond HGSOC.

Given the established role of DNMT1 in ovarian cancer (*66*, *67*), our findings linking METTL3/METTL14-mediated m^6^A modification to DNMT1 translation have important therapeutic implications. DNA methyltransferase inhibitors, including Food and Drug Administration (FDA)–approved agents such as 5-azacytidine and 5-aza-2′-deoxycytidine, have proven efficacy in hematologic malignancies. In this study, we demonstrated that the DNMT1 inhibitor GSK3484862 exerts antitumor activity in HGSOC, suppressing proliferation, migration, and inducing genome-wide hypomethylation. Notably, the biological effects of GSK3484862 were substantially stronger than those produced by shRNA-mediated DNMT1 depletion, despite regulating common downstream targets such as TNFAIP3 and FBXO32, suggesting that the extent of DNMT1 inhibition is a critical determinant of cellular response.

In conclusion, our study identifies DNMT1 as a translationally regulated m^6^A target downstream of METTL3 and METTL14 and establishes an m^6^A-METTL3-DNMT1 regulatory axis that enhances HGSOC progression. Although DNMT1 mediates specific METTL3-dependent functions, including tumor suppressor gene repression and cell migration, additional m^6^A-regulated targets likely contribute to the broader effects of METTL3 on proliferation and survival. These findings reveal a previously unrecognized mechanism linking m^6^A-dependent translational control to DNMT1-dependent gene repression and highlight the therapeutic potential of targeting both epitranscriptomic and epigenetic pathways in ovarian cancer.

## MATERIALS AND METHODS

### Datasets

To examine *METTL3*, *METTL14*, *DNMT1*, *TNFAIP3*, and *FBXO32* expression in patient samples, the GSE37582 dataset from the GEO database was analyzed by comparing the mRNA expression profiles of 74 ovarian cancer cases with 47 healthy controls. In addition, TCGA datasets were used to assess the correlation between *METTL3*, *METTL14*, *DNMT1*, *TNFAIP3*, and *FBXO32* expression levels and overall survival in patients with ovarian cancer.

### Cell culture, treatment, and siRNA transfection

OVCAR8 and OVCAR4 cells were grown in RPMI 1640 medium supplemented with 10% fetal bovine serum (FBS) and 1% penicillin/streptomycin (Gibco). HEK293T, MDA-MB-231, MiaPaCa2, SUIT2, and U2OS cells were cultured in Dulbecco’s modified Eagle’s medium (DMEM) (Gibco) supplemented with 10% FBS and 1% penicillin/streptomycin. For MDA-MB-231 and MiaPaCa2, media were additionally supplemented with human insulin (10 μg/ml; Sigma-Aldrich) and 2.5% horse serum, respectively. All the cells were maintained at 37°C and 5% CO_2_ in a humidified incubator. For METTL3 inhibition, cells were treated with STM2457 (MedChemExpress; HY-134836, 10 μM) for 48 hours and for 6 days for methylation array analysis. For DNMT1 inhibition, cells were treated with GSK3484862 (MedChemExpress; HY-135146, 10 μM) for 48 hours. To assess RNA stability, cells were treated with actinomycin D (5 μg/ml) for specified times, followed by RNA extraction. To assess protein stability, cells were treated with cycloheximide (CHX; 50 μg/ml) for specified times, followed by Western blot analysis. For coinhibition of TNFAIP3 and FBXO32, cells were transfected in 6-well plates with 100 nM each MISSION esiRNA targeting human FBXO32 (EHU155831) and TNFAIP3 (EHU019961) (Sigma-Aldrich) or with nontargeting control siRNA, using INTERFERin (Polyplus) transfection reagent.

### Antibodies

For Western blot analysis, the following antibodies were used at their respective dilutions: Anti-METTL3 (Abcam, ab221795, 1:1000), Anti-METLL14 (Sigma-Aldrich, HPA038002, 1:1000), Anti-KIAA1429 (Abcam, ab50993, 1:1000), Anti-CBLL1 (Abcam, ab50993, 1:2000), Anti-WTAP (Invitrogen, OTI12G, 1:2000), Anti-DNMT1 (Abcam, ab188453, 1:1000), Anti-UHRF1 (Thermo Fisher Scientific, PAF-29884, 1:1000), Anti-MAFbx/FBXO32 (Santa Cruz Biotechnology, sc-166806, 1:250), and Anti-A20/TNFAIP3 Antibody (4H16) (Santa Cruz Biotechnology, sc-69980, 1:500). The secondary antibodies included goat Anti-Rabbit IgG H&L [horseradish peroxidase (HRP)] (Abcam, ab6721, 1:5000) and goat Anti-Mouse IgG H&L (HRP) (Abcam, 1:1000, ab6789). Anti-β-actin (Sigma-Aldrich, A5441, 1:2000) or β-tubulin (Sigma-Aldrich, T2200, 1:2000) were used as loading controls for Western blotting. For immunofluorescence (IF) staining, the following antibodies were used: Anti-METTL3 (Abcam, ab221795, 1:250), Anti-METLL14 (Sigma-Aldrich, HPA038002, 1:250), Anti-KIAA1429 (Abcam, ab50993, 1:100), Anti-CBLL1 (Abcam, ab50993, 1:250), Anti-WTAP (Invitrogen; OTI12G, 1:100), Anti-DNMT1 (Abcam; ab188453, 1:250), and Anti-5mC (Active Motif; 39649, 1:250). The secondary antibodies used for IF staining included goat anti-rabbit IgG AF568 (Thermo Fisher Scientific, A11011, 1:1000) and goat anti-mouse IgG AF488 (Thermo Fisher Scientific, A11029, 1:1000).

### Human ovarian cancer specimens and immunohistochemistry

To investigate the correlation between the expression of METTL3, DNMT1, TNFAIP3, and FBXO32 in patient samples, 11 HGSOC tissues along with 6 normal ovarian tissues were used in the study (table S1). For protein extraction, tissues were finely minced and suspended in 300 μl of lysis buffer containing 50 mM Hepes (pH 7.5), 150 mM NaCl, 3 mM MgCl_2_ 0.2% Triton X-100, 0.2% Nonidet P-40, and 10% glycerol, supplemented with a protease inhibitor cocktail. The tissues were homogenized using a tissue homogenizer and centrifuged at 12,000 rpm for 15 min. The resulting supernatant containing total protein was quantified using the standard Bradford assay and used for immunoblotting. IHC analysis was performed on ovarian cancer TMAs obtained from the company Tissue Array (OV1006 and OV802c819) (https://tissuearray.com/) using a primary antibody against DNMT1 (Abcam, ab188453). The OV1006 TMA consists of 100 cores representing 100 individual cases, including 62 serous carcinomas, 10 mucinous adenocarcinomas, 5 clear cell carcinomas, 3 endometrioid adenocarcinomas, 10 lymph node metastases, and 10 adjacent normal ovarian tissue samples. The OV802c819 TMA comprised 40 serous carcinomas, 6 clear cell carcinomas, 2 mucinous adenocarcinomas, 1 stromal tumor, 2 sex cord-stromal tumors, 9 germ cell tumors, 10 metastatic adenocarcinomas, and 10 adjacent normal ovarian tissue samples. Although both TMAs included adjacent normal ovarian tissues, epithelial cells were not present in these sections; therefore, the normal samples were excluded from the final analysis. DNMT1 expression was quantified using the *H* score, calculated as the product of staining intensity and percentage of positively stained cells. The staining intensity was graded on a scale from 0 to 3 (0: no staining; 1: weak; 2: moderate; and 3: strong), whereas the percentage of positive cells was scored from 0 to 4 (0: 0%; 1: 1 to 25%; 2: 26 to 50%; 3: 51 to 75%; and 4: 76 to 100%).

### Generation of *METTL3*, *METTL14*, and *DNMT1* knockdown OVCAR8 cells

Lentiviral particles were produced by transfecting HEK293T cells with the packaging vector pCMVdR8.2, the helper plasmid pCMV-VSV-G, and pLKO.1-puro plasmids containing shRNAs targeting *METTL3*, *METTL14*, *DNMT1*, or a Scr control sequence (target sequences provided in table S2). Transfections were carried out using jetPEI (Polyplus) according to the manufacturer’s protocol or CaCl_2_. Lentiviral particles containing the supernatant were harvested 48 and 72 hours posttransfection. After 48 hours of supernatant collection, the cells were supplemented with fresh medium. Lentiviral particles were concentrated using Amicon Ultra-15 Centrifugal Filter Units (Merck, Darmstadt, Germany). OVCAR8 cells were transduced with Scr, *METTL3*, and *METTL14* shRNA containing lentiviral particles in the presence of polybrene (8 μg/ml). The transduced cells were selected with puromycin (0.1 μg/ml) for 2 days postinfection.

### Cell proliferation assay

A total of 2 × 10^4^ cells, depleted of METTL3, METTL14, and DNMT1 and treated with either STM2457 or GSK3484862 as indicated, were seeded into 6-well plates. The cell numbers were quantified on days 2, 4, and 6 after seeding. Proliferation rates were calculated and normalized to Scr, and relative growth curves were plotted. As an alternative approach, cell proliferation was assessed using the MTT assay. Briefly, 3 × 10^3^ cells were seeded into each well of a 96-well plate and allowed to adhere overnight. MTT reagent (5 mg/ml) was added at 24, 48, 72, and 96 hours after seeding. Cells were incubated with MTT for 4 hours at 37°C to allow the formation of formazan crystals. Subsequently, the culture medium containing MTT was carefully aspirated, and the resulting formazan crystals were dissolved in 100 μl of DMSO. Absorbance was measured at 570 nm using a microplate reader. Cell proliferation was determined on the basis of the absorbance values and expressed relative to the 24 hours of each experimental group.

### Colony formation assay

Cells depleted of METTL3 or METTL14 and treated with STM2457 or GSK3484862 as indicated, were seeded at low density (1 × 10^3^ cells per well) in 6-well plates for colony formation assays. The cells were cultured for 10 days, and the medium was replenished every 48 hours. The colonies were fixed with 10% formaldehyde and stained with 0.25% crystal violet. Plates were digitally imaged, and the crystal violet–stained colonies were solubilized in 10% acetic acid for quantification. The absorbance was measured at 570 nm using a microplate reader (Tecan, Thermo Fisher Scientific). Colony growth was expressed as the percentage of cell viability relative to Scr.

### Transwell migration assay

Cells depleted of METTL3, METTL14, or DNMT1 and treated with STM2457 or GSK3484862 as indicated were cultured in serum-free growth medium for 48 hours to induce cell cycle arrest and minimize proliferation. Subsequently, 5 × 10^5^ serum-starved cells were seeded into the upper chambers of 12-well transwell inserts (Corning), whereas the lower chambers were filled with growth medium containing 10% FBS to serve as a chemoattractant. After 24 hours of migration, the cells on both sides of the membrane were fixed with 10% formaldehyde and stained with 0.25% crystal violet. Nonmigrating cells on the upper surface of the membrane were removed using a sterile cotton swab. The migrated cells on the lower membrane surface were visualized using a Leica microscope at 20× magnification. For quantification, migrated cells were counted in six random fields per membrane using the ImageJ Cell Counter plugin. Data are plotted as the average number of migrated cells relative to Scr.

### Cell cycle and apoptosis assay

Cell cycle and apoptosis analyses were conducted using the BD Accuri C6 Plus Flow Cytometer and the Muse Cell Analyzer (Millipore, Sigma-Aldrich), respectively, following the manufacturer’s protocols. For cell cycle analysis using BD Accuri C6 Plus, the cells were stained with propidium iodide (PI; Thermo Fisher Scientific). For apoptosis assays, Annexin V Ready Flow Conjugates for Apoptosis Detection (Thermo Fisher Scientific) were used in combination with eBioscience 7-AAD Viability Staining Solution, according to the manufacturer’s instructions.

### Quantitative real-time PCR

Total RNA was extracted using the RNeasy Plus Mini Kit (Qiagen) or TRIzol reagent, as previously described (*68*). Equal amounts of RNA were used for cDNA synthesis using the RevertAid First Strand cDNA Synthesis Kit (Invitrogen), following the manufacturer’s protocol. Quantitative real-time PCR (qRT-PCR) was performed using gene-specific primers (table S2) and SYBR Green Master Mix (Thermo Fisher Scientific) on the QuantStudio Real-Time PCR System (Applied Biosystems). β-Actin served as an internal control for normalization. Cycle threshold (CT) values were analyzed using the 2^−ΔΔCT^ method to calculate the relative fold changes across at least three independent biological replicates.

### Immunoblotting

Whole-cell lysates were prepared by incubating the cells in lysis buffer containing 50 mM Hepes (pH 7.5), 150 mM NaCl, 3 mM MgCl_2_ 0.2% Triton X-100, 0.2% Nonidet P-40, and 10% glycerol, supplemented with a protease inhibitor cocktail. Proteins were resolved on 8 to 10% SDS–polyacrylamide gel electrophoresis (PAGE) gels and transferred to polyvinylidene difluoride (PVDF) membranes (0.45 μm, Merck Millipore). Membranes were blocked with 5% skim milk (Invitrogen) for 1 hour at room temperature, followed by overnight incubation with primary antibodies at 4°C. After washing, the membranes were incubated with HRP-conjugated secondary antibodies for 1 hour. Protein bands were visualized using enhanced chemiluminescence (Thermo Fisher Scientific) and imaged using an Amersham Imager 680.

### Immunofluorescence

METTL3-depleted cells were seeded onto poly-l-lysine–coated coverslips in 12-well plates, fixed with 4% formaldehyde (Sigma-Aldrich), and permeabilized using 0.25% Triton X-100 (Sigma-Aldrich). Following two washes with phosphate-buffered saline (PBS), the cells were blocked for 1 hour at room temperature in blocking solution containing 10% goat serum (Invitrogen), 1% bovine serum albumin (HyClone), and 0.01% Tween 20 (Sigma-Aldrich). Subsequently, cells were incubated with METTL3 and METTL14 primary antibodies overnight at 4°C. After washing with PBS, cells were incubated with fluorescence-conjugated secondary antibodies. Nuclei were counterstained with 4′,6-diamidino-2-phenylindole (DAPI). Fluorescence images were acquired using a Leica SP8 confocal microscope and analyzed using the ImageJ software.

### RNA-seq and differential gene expression analysis

RNA-seq for METTL3 and METTL14 knockdown was performed using Novogene–Advancing Genomics, Improving Life (https://en.novogene.com/) using two biological replicates. RNA-seq analysis of GSK3484862-treated OVCAR8 cells was conducted by Biomarker Technologies (BMKGENE) (www.bmkgene.com) with three biological replicates. DEGs were identified from the combined dataset using a log_2_ fold change threshold (up-regulation > 0.58; down-regulation < −0.58) and a significance cutoff of adjusted *P* (*P*_adj_) < 0.05 [−log_10_(*P*_adj_) > 1.3].

### GO analysis

GO analysis of DEGs was performed using the web-based tool Database for Annotation, Visualization, and Integrated Discovery (DAVID).

### Xenograft study

Luciferase-labeled OVCAR8 cells (1 × 10^6^) were subcutaneously injected into 6- to 7-week-old female Crl:NU(NCr)-Foxn1^nu^ nude mice. Images at day 0 were taken immediately after cell injection. In vivo bioluminescence imaging was performed using a PerkinElmer IVIS system (model INVCLS136335) with the Living Image software (v4.3.1). For imaging, luciferin (150 μg/ml, 100 μl) was retro-orbitally injected into the mice. Tumor volume was measured with a digital caliper using the following formula: tumor volume = 1/2 × (length × width^2^). Tumors were collected at the experimental endpoint for further analysis.

### Methylated RNA immunoprecipitation sequencing

MeRIP-seq was performed as previously described (*69*). Briefly, fragmented mRNA (5 μg) was immunoprecipitated overnight with m^6^A antibody (10 μg, Abcam, ab151230). Protein A Dynabeads (61006, Thermo Fisher Scientific) was used to capture the m^6^A-modified mRNA. The beads were then washed, and the pool of m^6^A-modified transcripts was eluted in 16 μl of diethyl pyrocarbonate (DEPC)–treated water. Four microliters of the eluate was used to prepare cDNA using the SuperScript VILO cDNA Synthesis Kit (Thermo Fisher Scientific), and qRT-PCR was performed for *GAPDH* and *MYC* to validate the immunoprecipitation. Ten microliters (∼100 ng) of the eluate was sent to Novogene (https://en.novogene.com/) for library preparation and sequencing. Integrative Genomics Viewer (IGV) was used to study the m^6^A peaks of specific mRNAs.

### SELECT

Site-specific m^6^A detection of the DNMT1 transcript was performed using a single-base elongation– and ligation-based qPCR amplification method (SELECT), as previously described (*26*). Up/down probes were designed on the basis of the MeRIP-seq coordinates of m^6^A and m^6^A-6 sites (as input) on DNMT1. Briefly, mRNA was purified from total RNA extracted from DMSO- and STM2457-treated and METTL3- and METTL14-depleted OVCAR8 cells using Protein A Dynabeads (61006, Thermo Fisher Scientific). The mRNA was first annealed with the respective up/down probes using dNTPs (N0447S, NEB) and CutSmart buffer (B6004S, NEB) by incubating at the following decreasing temperature gradient: 90°C for 1 min, 80°C for 1 min, 70°C for 1 min, 60°C for 1 min, 50°C for 1 min, and 40°C for 6 min in a thermocycler (Applied Biosystems). The m^6^A-dependent gap filling and ligation were facilitated by incubating annealed samples with Bst 2.0 DNA Polymerase (M0537S, NEB), SplintR ligase (M0375S, NEB), and Adenosine 5′-Triphosphate (ATP) (P0756S, NEB) in a thermocycler for 20 min at 40°C, followed by 20 min at 80°C. SELECT products were quantified by qRT-PCR using primers targeting a small overhang sequence present on each up/down probes. ΔCT values were calculated by normalizing the Ct value for each m^6^A site to its input. ΔΔCT values were obtained by normalizing STM2457-treated samples to the ΔCT of DMSO controls. The relative SELECT products were calculated by inverting the log transformation using the 2^−ΔΔCT^. The sequences of the probes and primers are listed in table S2.

### Polysome profiling

METTL3-depleted and Scr control cells were treated with CHX (100 μg/ml) for 5 min and washed twice with cold 1x PBS containing CHX to halt translation. Equal numbers of cells were lysed in polysome lysis buffer [20 mM tris-HCl (pH 7.4), 5 mM MgCl_2_, 150 mM NaCl, and 1% Triton X-100] supplemented with CHX (100 μg/ml), dithiothreitol (1 mM), protease inhibitors (complete EDTA-free, Roche), and RNase Inhibitor (200 U/ml; Thermo Fisher Scientific). Lysates were agitated on a rocking platform for 10 min and then centrifuged at 10,000*g* for 10 min at 4°C. Equal volumes of clarified lysates were layered onto sucrose gradients [15 to 45% (w/v)] that were previously prepared using a Gradient Master (BioComp Instruments). The lysates were centrifuged at 40,000*g* for 2 hours at 4°C in an ultracentrifuge with an SW60Ti rotor (Beckman). Fractions were collected using a Gilson Fraction FC-203B collector in Eppendorf tubes containing 300 μl of solution II [10 mM tris-HCl (pH 7.5), 350 mM NaCl, 10 mM EDTA, 1% SDS, and 42% urea] while measuring the absorbance at 260 nm using a Triax flow cell (BioComp Instruments). Polysome profiles were generated by plotting absorbance against piston position.

### Northern blotting

Total RNA was isolated from METTL3-depleted and Scr control cells using TRIzol, without DNase treatment, and quantified using a NanoDrop spectrophotometer. Equal amounts of RNA (4 μg) per sample was aliquoted for gel loading. RNA samples were denatured in a buffer containing 1X Mops, 2.2 M formaldehyde, and 50% formamide at 65°C for 12 min, cooled on ice for 2 min, and mixed with loading dye [50% glycerol, 1 mM EDTA (pH 8.0), 0.01% bromophenol blue, and 0.01% xylene cyanol] supplemented with ethidium bromide (0.5 μg/ml). A 1.5% agarose gel containing 2.2 M formaldehyde in 1X Mops buffer [20 mM Mops, 5 mM sodium acetate, and 1 mM EDTA (pH 7.0)] was prepared and allowed to solidify for at least 1 hour. Samples were electrophoresed in 1X Mops running buffer at 100 V until the dye front reached the bottom of the gel (∼5 hours). Following electrophoresis, the gel was washed twice in sterile Milli-Q water for 10 min each and imaged and then incubated in alkaline solution (0.05 M NaOH and 0.3 M NaCl) for 20 min, washed three times in sterile water (5 to 10 min each), and soaked in 20X SSC for at least 30 min. RNA was transferred onto a Hybond-N+ membrane by capillary blotting in 20X SSC for 24 to 48 hours, followed by ultraviolet cross-linking. The membrane was prehybridized in ROTIHybri-Quick (A981.1, Roth) at 52°C for 60 min and then hybridized overnight at 52°C with a radiolabeled oligonucleotide probe. The sequences of the probes are listed in table S2. The probe was generated by incubating 1 μM oligonucleotide with 1X T4 PNK buffer, 1 U T4 PNK, and [γ-^32^P]ATP at 37°C for 50 min, followed by purification using an oligo clean-up kit and elution in TE buffer. After hybridization, the membrane was washed sequentially in 2X SSC with 0.5% SDS for 30 min at 37°C and in 0.5X SSC with 0.05% SDS for 20 min at 37°C and then rinsed in 2X SSC and air dried. The membrane was wrapped and exposed to x-ray film for 4 hours, and the resulting film was imaged using a phosphorimager.

### Luciferase reporter assay

The DNMT1 Exon 8 sequence was amplified by PCR (Phusion High-Fidelity DNA Polymerase, Thermo Fisher Scientific) from cDNA generated from OVCAR8 cell RNA using the RevertAid kit (Thermo Fisher Scientific) with oligo(dT) primers. The resulting amplicon was digested with Xho I and Not I and cloned into the multiple cloning site of a modified psiCHECK2 vector, in which all consensus m^6^A motifs within the Renilla and firefly luciferase CDSs had been ablated to eliminate confounding effects from endogenous methylation of the reporter backbone. The vector was digested with the same restriction enzymes and gel purified using the QIAquick Gel Extraction Kit (Qiagen) before ligation. The resulting DNMT1 Exon 8–psiCHECK2 construct was verified by Sanger sequencing. Primer and sequence information for both constructs are provided in table S2. The DNMT1 Exon 8–psiCHECK2 construct was mutated for the m^6^A site (A-to-C) using Gibson cloning. For the luciferase assay, OVCAR8 cells were seeded in 12-well plates and transfected with the WT DNMT1 Exon 8–psiCHECK2 reporter, the m^6^A site-mutated DNMT1 Exon 8–psiCHECK2 reporter, or an empty vector, using Lipofectamine LTX according to the manufacturer’s instructions (Thermo Fisher Scientific). At 24 hours posttransfection, cells transfected with either the WT or mutant reporter were treated with the METTL3 inhibitor STM2457 or DMSO (vehicle control). Dual-luciferase assays were performed 48 hours after treatment using the Dual-Luciferase Reporter Assay System (Promega) per the manufacturer’s protocol. Renilla luciferase activity was normalized to firefly luciferase activity for each sample. Relative luciferase activity was calculated for STM2457-treated versus DMSO-treated cells for both the WT and m^6^A site-mutated reporters, allowing comparison of STM2457 sensitivity between the two constructs.

### MethylationEPIC v2.0 BeadChip hybridization

DNA bisulfite conversion and array hybridization were conducted at the Genomics Unit of Josep Carreras Leukemia Research Institute (Badalona, Spain). Total DNA was quantified using the Qubit dsDNA Broad Range Assay Kit (Invitrogen, Thermo Fisher Scientific, MA, USA), according to the manufacturer’s instructions. Subsequently, 600 ng of DNA was bisulfite converted using the EZ-96 DNA Methylation Kit (Zymo Research Corp., CA, USA) following the manufacturer’s protocol. Last, 200 ng of bisulfite-converted DNA was hybridized onto the Infinium HumanMethylationEPIC version 2.0 BeadChip microarray (Illumina Inc.), as previously described in (*70*).

### DNA methylation analysis

All analyses were conducted using the R statistical software (R version 4.1.2) (www.R-project.org), following the minfi pipeline (*71*). Briefly, raw signal intensity files (IDATs) were processed, and sample quality was evaluated by excluding samples with a mean detection *P* value of ≥0.05. Next, normalization was performed using the Noob method, and low-quality probes were filtered out. Specifically, probes with a mean detection *P* value of ≥0.01 in more than 10% of samples, cross-reactive probes, as well as those associated with sex chromosomes and SNPs, were removed from the analysis. Probes with missing values were imputed on the basis of the mean methylation values of the corresponding probes from other samples. Last, methylation beta values and *M* values were generated for downstream analysis. Unsupervised hierarchical clustering was performed using all CpG probes to cluster the samples using the Euclidean distance and the ward. D2 clustering method. For row clustering, a random subset comprising 1% CpG probes was selected to reduce computational complexity. A heatmap was generated and visualized using the ComplexHeatmap package in R (*72*). t-SNE analysis was performed on all CpG sites in the array using the Rtsne R package (*73*). Global methylation analysis was performed by calculating the mean methylation value for each sample, which was then plotted and grouped by condition using the ggplot2 R package (*74*). To identify differentially methylated probes, a linear regression model was applied to methylation *M* values, and one condition was compared. CpG sites were considered statistically significant if the adjusted *P* value was ≤0.05. In addition, a biological filter was applied, considering only probes that were statistically significant and a mean methylation difference of at least |0.5| between the conditions. The annotation of differentially methylated probes was performed using the UCSC annotation (GRCh38/hg38). Gene set enrichment analysis was performed using the EnrichR R package (*75*) for genes associated with differentially methylated CpG sites identified in the previous analysis. The analysis was performed using GO databases including “Biological Process 2023,” “Molecular Function 2023,” and “Cellular Component 2023.” A gene set was considered statistically significant if the adjusted *P* value was ≤0.05.

### Statistical analysis

All data are shown as mean ± SD. Graphs were plotted, and statistical analyses were performed using GraphPad Prism (version 10). Student’s *t* test and one-way analysis of variance (ANOVA) were used to calculate significance. *P < 0.05, **P < 0.01, ***P < 0.001, and ****P < 0.0001 were considered as statistically significant.

### Ethics statement

All ovarian cancer specimens were collected with informed consents from the patients from the biobank of Norrlands Universitetssjukhus, Umeå, Sweden. All samples were handled in accordance with the approved biobank protocol (Dnr: 2019-03729). The Ethics Committee for Animal Experimentation (CEEA-PRBB) had approved all animal experiments conducted at the Hospital del Mar Medical Research Institute, Barcelona, Spain.
