# Supplementary material for: METTL3-mediated m6A modification of DNMT1 enhances ovarian cancer progression
Source: Sci Adv. 2026 Nov 4;12(45):eaec2297. doi: 10.1126/sciadv.aec2297 (PMC13733898; doi:10.1126/sciadv.aec2297)
Supplement: Supplementary file 1 [file sciadv.aec2297_sm.pdf]

Science Advances
AAAS

Supplementary Materials for

METTL3-mediated m^{6}A modification of DNMT1 enhances ovarian cancer progression

Kanchan Kumari *et al.*

Corresponding author: Kanchan Kumari, kanchan.kumari@umu.se; Francesca Aguilo, francesca.aguilo@umu.se

Sci. Adv. **12**, eaec2297 (2026)
DOI: 10.1126/sciadv.aec2297

The PDF file includes:

Figs. S1 to S7
Legends for data S1 to S4
Tables S1 and S2

Other Supplementary Material for this manuscript includes the following:

Data S1 to S4

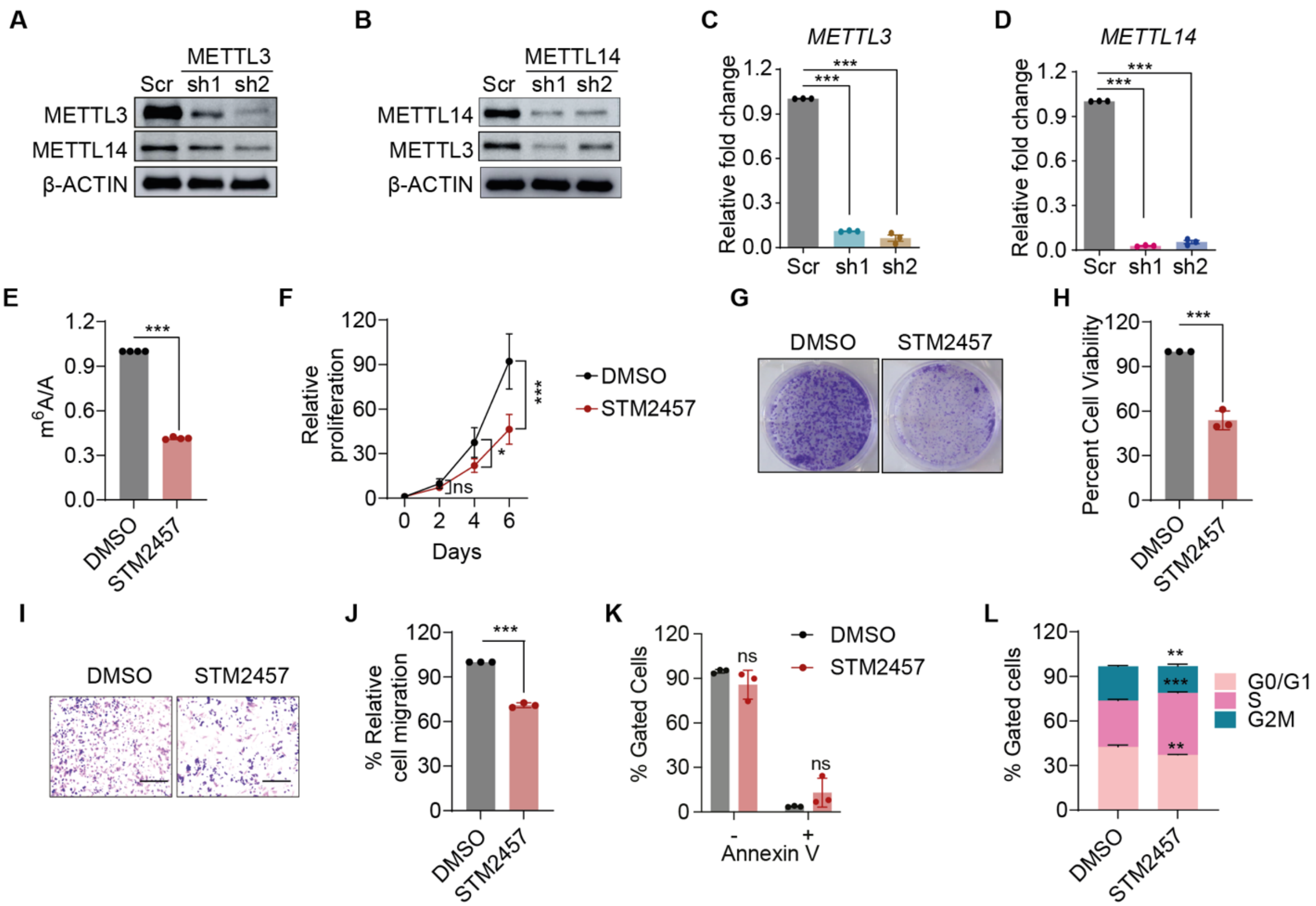

fig. S1. Knockdown efficiency of METTL3 and METTL14 and the effect of pharmacological inhibition of METTL3 in OVCAR8. (**A** and **B)** Representative Western blot images showing knockdown efficiency of METTL3 (**A**) and METTL14 (**B**) β-ACTIN serves as a loading control. (**C** and **D)** qRT-PCR analysis of *METTL3* (**C**) and *METTL14* (**D**) mRNA knockdown efficiency; *β-actin* serves as a reference gene for normalization. **(E)** Levels of m^6A upon STM2457 treatment as measured by quantitative LC-MS/MS. **(F)** Line graph depicting the growth of DMSO and STM2457-treated cells over a 6-day period. **(G)** Representative images from the colony formation assay conducted over 10 days, demonstrating the ability of OVCAR8 cells to form colonies in the presence of DMSO or STM2457. **(H)** Bar graph depicting relative percent cell viability calculated from absorbance values at 570 nm of dissolved crystal violet, corresponding to the colony formation assay in panel (**G**). (**I**) Representative trans-well assay images illustrating the migration of DMSO- or STM2457-treated cells. Scale bar, 200 µm. (**J**) Quantification of migrated cells from panel (**I**), calculated from absorbance values at 570 nm of dissolved crystal violet stain. **(K)** Bar graph displaying the percentage of live (Annexin V–) and apoptotic (Annexin V+) cells in DMSO- and STM2457-treated OVCAR8 cells. (**L**) Cell cycle distribution of cells following STM2457 treatment. Data are presented as mean ± SD or as representative images of n > 3 independent biological experiments. Statistical analysis: Two-tailed Student's *t*-test (**C**, **D, E, F, H, J, K, L**). Data are mean ± s.d.; n = 3(**A** to **H, K** and **L**), n=2(**I** and **J**) independent biological experiments.

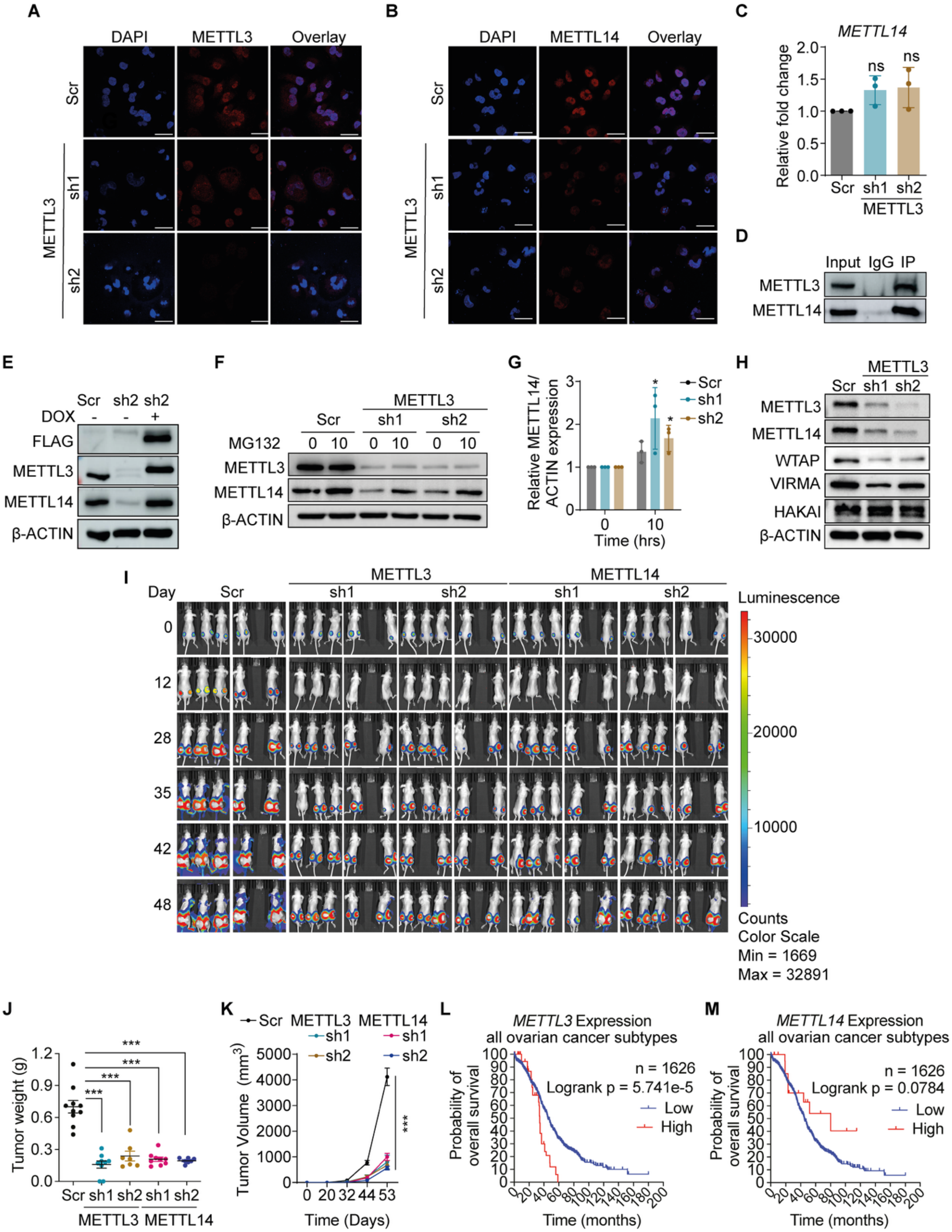

Figure legend on the next page.

fig. S2. METTL3 regulates METTL14 protein stability and co-depletion of METTL3 and METTL14 suppresses tumor growth in vivo. (A and B) Immunofluorescence images of METTL3 (A) and METTL14 (B) expression in METTL3-depleted cells. **(C)** qRT-PCR of METTL14 following METTL3 knockdown; β-actin as reference gene. **(D)** Western blot of METTL14-METTL3 interaction following METTL3 immunoprecipitation; rabbit IgG as negative control. **(E)** Western blot of FLAG, METTL3, and METTL14 levels in Scr, sh2METTL3, and sh2METTL3 + Dox-inducible FLAG-METTL3 cells. **(F)** Western blot of METTL3 and METTL14 in METTL3-depleted cells following MG132 treatment for 10 hours. **(G)** Normalized METTL14 protein levels from (F). **(H)** Western blot of METTL3, METTL14, WTAP, VIRMA, and HAKAI upon METTL3 knockdown. **(I)** Representative bioluminescence images of nude mice subcutaneously transplanted with luciferase-labelled Scr, METTL3-depleted (sh1 and sh2), or METTL14-depleted (sh1 and sh2) OVCAR8 cells at days 0, 12, 28, 35, 42, and 48 post-injection. Color scale indicates signal intensity (red = highest, blue = lowest). **(J)** Tumor weights from Scr, METTL3-, and METTL14-depleted OVCAR8 cells corresponding to Fig. 1O. **(K)** Growth of Scr and METTL3- and METTL14-depleted OVCAR8 tumors over time. **(L and M)** Kaplan-Meier overall survival curves for METTL3 (L) and METTL14 (M) across ovarian cancer subtypes (TCGA). β-ACTIN served as the western blot loading control. Statistical analysis: Two-tailed. Student's *t*-test (**C**, **G**). one-way ANOVA with Dunnett's correction for multiple comparison (**J, K**). Data are mean ± s.d.; n = 3(**A** to **H**), independent biological experiments.

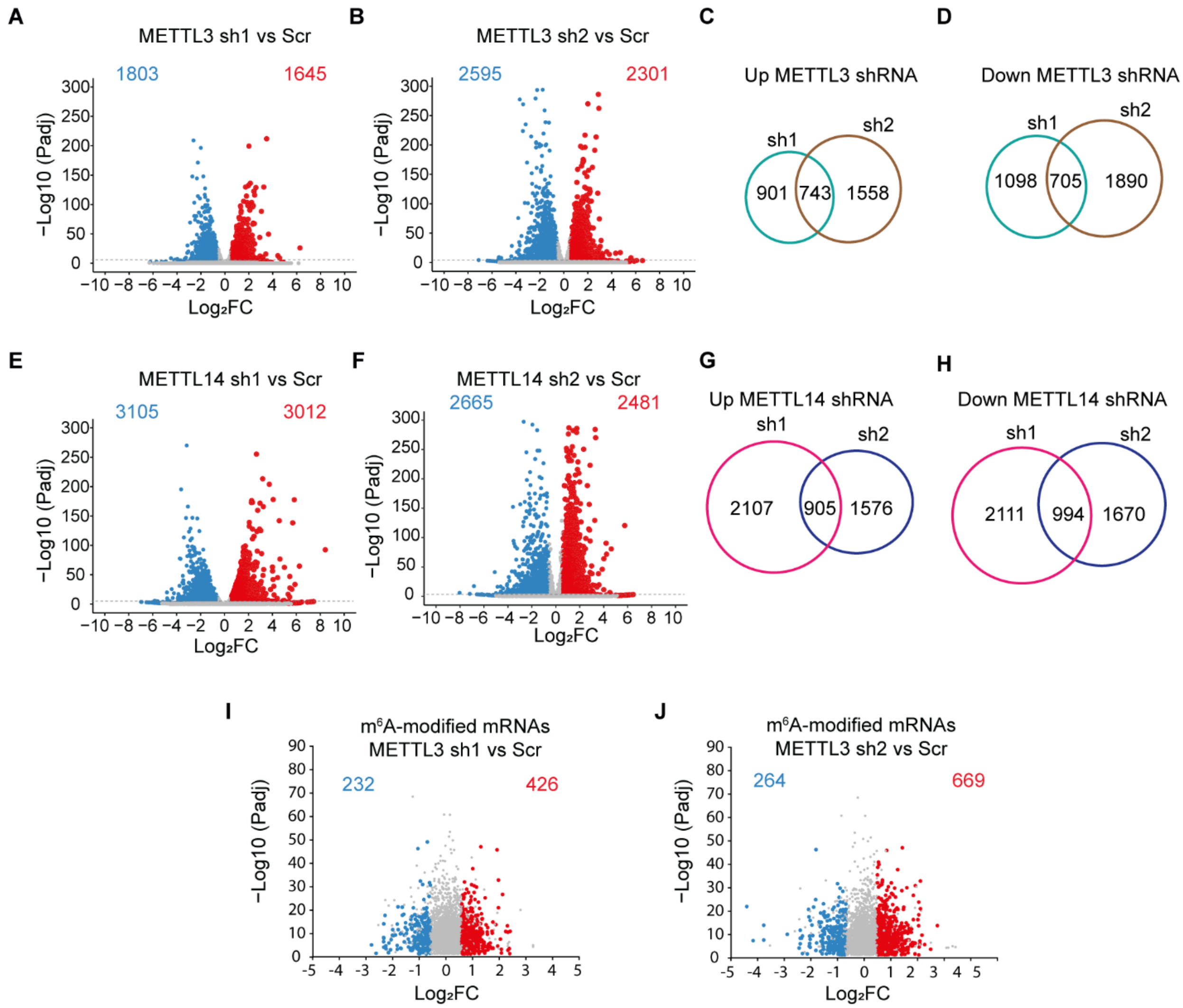

fig. S3. Differentially expressed genes upon METTL3 and METTL14 depletion and their overlap with m^{6}A-modified transcripts. (A and B). Volcano plots of differentially expressed genes in sh1METTL3- (**A**) and sh2METTL3-depleted (**B**) OVCAR8 cells compared to Scr cells, highlighting significantly up- (red) and down-regulated (blue) genes. (**C** and **D)** Venn diagrams showing commonly upregulated (**C**) and downregulated (**D**) genes in METTL3-depleted OVCAR8 cells. (**E** and **F)** Volcano plots of differentially expressed genes in sh1METTL14- (**E**) and sh2METTL14-depleted (**F**) OVCAR8 cells compared to Scr cells, highlighting significantly up- (red) and down-regulated (blue) genes. (**G** and **H).** Venn diagrams showing commonly upregulated (**G**) and downregulated (**H**) genes in METTL14-depleted OVCAR8 cells. (**I** and **J).** Volcano plots showing m^{6}A-modified differentially expressed mRNAs upon METTL3 knockdown with sh1METTL3 (**I**) and sh2METTL3 (**J**). Representative images of n=2 independent biological experiments.

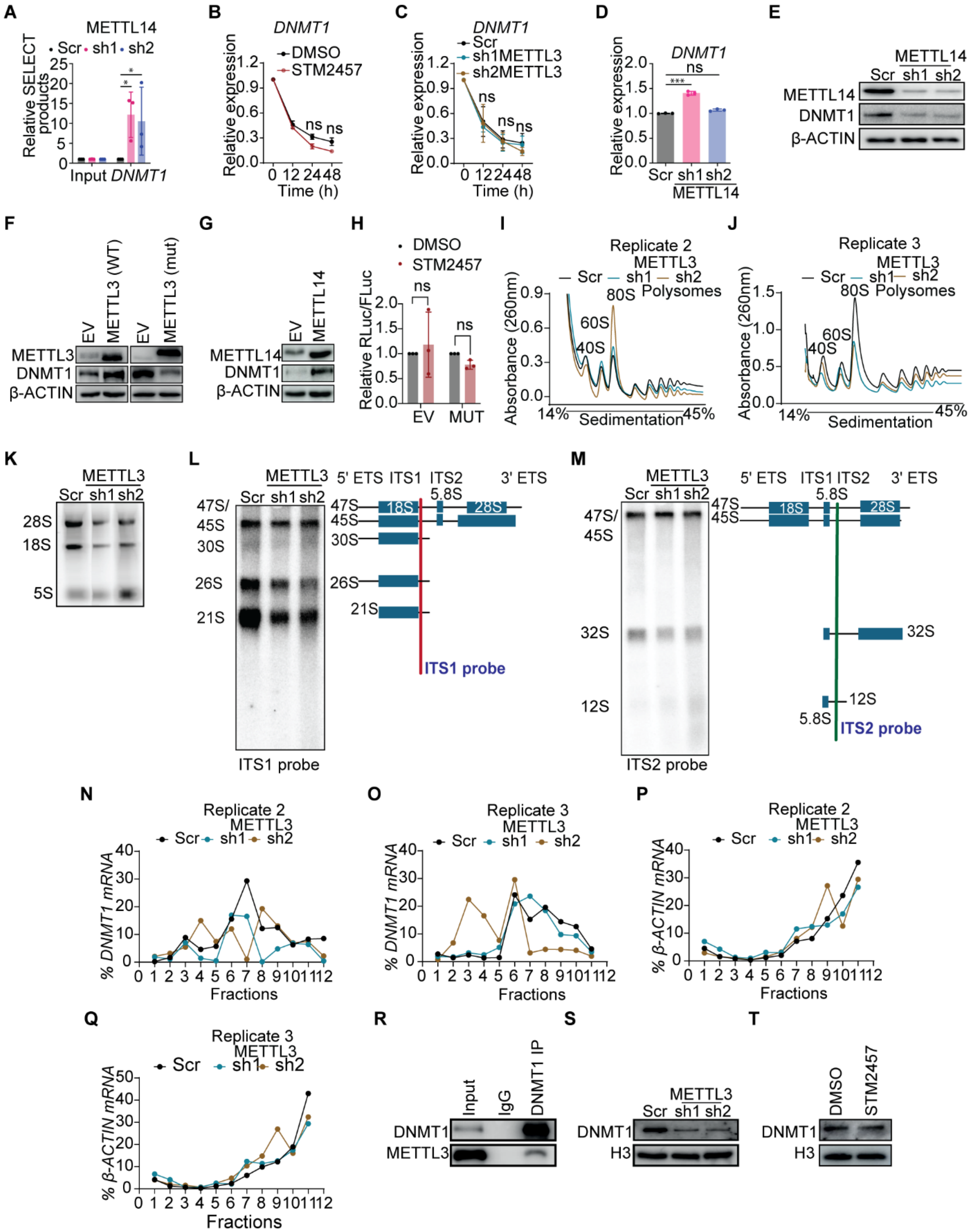

Figure legend on the next page.

fig S4. METTL14 regulates DNMT1 expression and mRNA stability, and METTL3 depletion impairs ribosome biogenesis, translation, and DNMT1 chromatin association in ovarian cancer cells. (A) Bar graphs of SELECT product levels upon METTL14 depletion. **(B and C)** DNMT1 mRNA stability following Actinomycin D treatment in STM2457-exposed (B) and METTL3-depleted (C) cells at 12, 24, and 48 hours. **(D)** qRT-PCR of DNMT1 expression upon METTL14 depletion; β-actin as reference gene. **(E)** Western blot of DNMT1 in METTL14-depleted cells. **(F and G)** Western blots of DNMT1, METTL3, or METTL14 upon overexpression of METTL3 WT or catalytic mutant (mut) (F) and METTL14 (G); EV: empty vector. **(H)** Relative RLuc/FLuc in OVCAR8 cells transfected with EV or m6A site-mutated psiCHECK2-DNMT1-Exon8 (MUT) following DMSO or STM2457 treatment. **(I and J)** Polysome profiling curves of METTL3-depleted cells showing global ribosomal distribution. **(K)** Agarose gel of 28S, 18S, and 5S/5.8S rRNA levels in Scr and METTL3-depleted (sh1 and sh2) OVCAR8 cells. **(L and M)** Northern blot of pre-rRNA processing intermediates in METTL3-depleted OVCAR8 cells using ITS1 (L) and ITS2 (M) probes end-labeled with [γ-32P] ATP. Pre-rRNA intermediates indicated in respective schematic diagrams. **(N to Q)** qRT-PCR of DNMT1 (N and O) and β-actin (P and Q) mRNA in polysome fractions from (I and J). **(R)** Co-immunoprecipitation of METTL3 following DNMT1 immunoprecipitation in OVCAR8 cells; rabbit IgG as negative control; input = 5% total lysate. **(S and T)** Western blot of DNMT1 in chromatin-bound fractions from Scr and METTL3-depleted (S) or STM2457-treated (T) cells; Histone H3 as loading control. β-ACTIN served as the western blot loading control. Statistical analysis: Two-tailed. Student's *t*-test (**A** to **D**, **H**). Data are mean ± s.d.; n = 3(**A** to **H, K** to **M** and **R** to **T**), independent biological experiments.

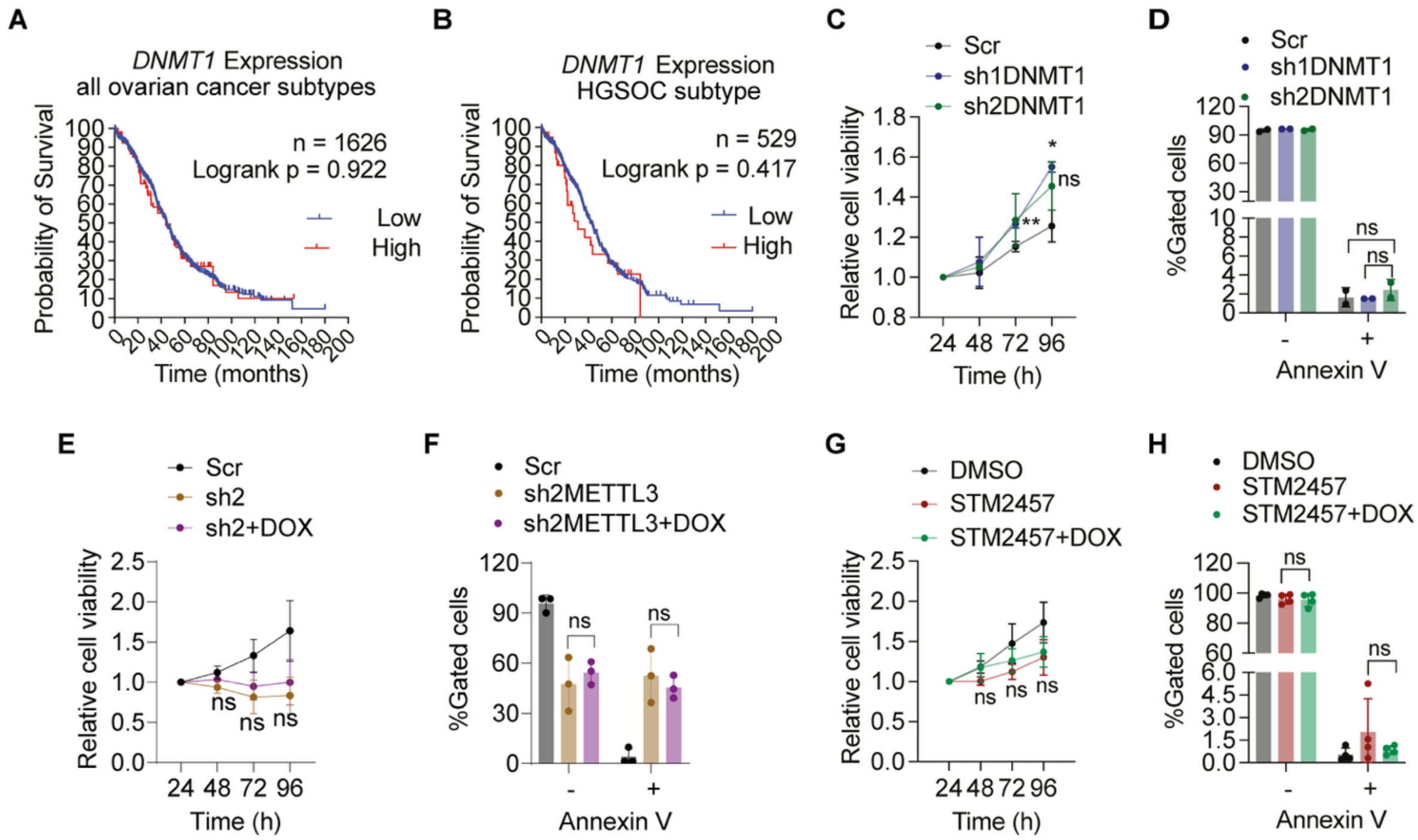

fig. S5. DNMT1 levels associate with HGSOC survival but do not significantly affect proliferation or apoptosis. (**A** and **B)** Kaplan-Meier survival curves showing the association between *DNMT1* expression and overall survival in TCGA datasets across all ovarian cancer subtypes (**A**) and in high-grade serous ovarian cancer (HGSOC) specifically (**B**). HR and log-rank *p* values are indicated. (**C** and **D)** Graphs showing the effect of DNMT1 knockdown on cell proliferation (**C**) and apoptosis (**D**). (**E** and **F)** Proliferation (**E**) and apoptosis (**F**) in Scr control, sh2METTL3, and sh2METTL3 + Dox-inducible FLAG-DNMT1 expressing OVCAR8 cells. (**G** and **H)** Graphs showing cell proliferation (**G**) and apoptosis (**H**) in DMSO control, STM2457-treated, and STM2457 + Dox-inducible FLAG-DNMT1 expressing OVCAR8 cells. Two-tailed Student's *t*-test (**C** to **H**). Data are mean ± s.d.; n = 3(**C** to **H**), independent biological experiments.

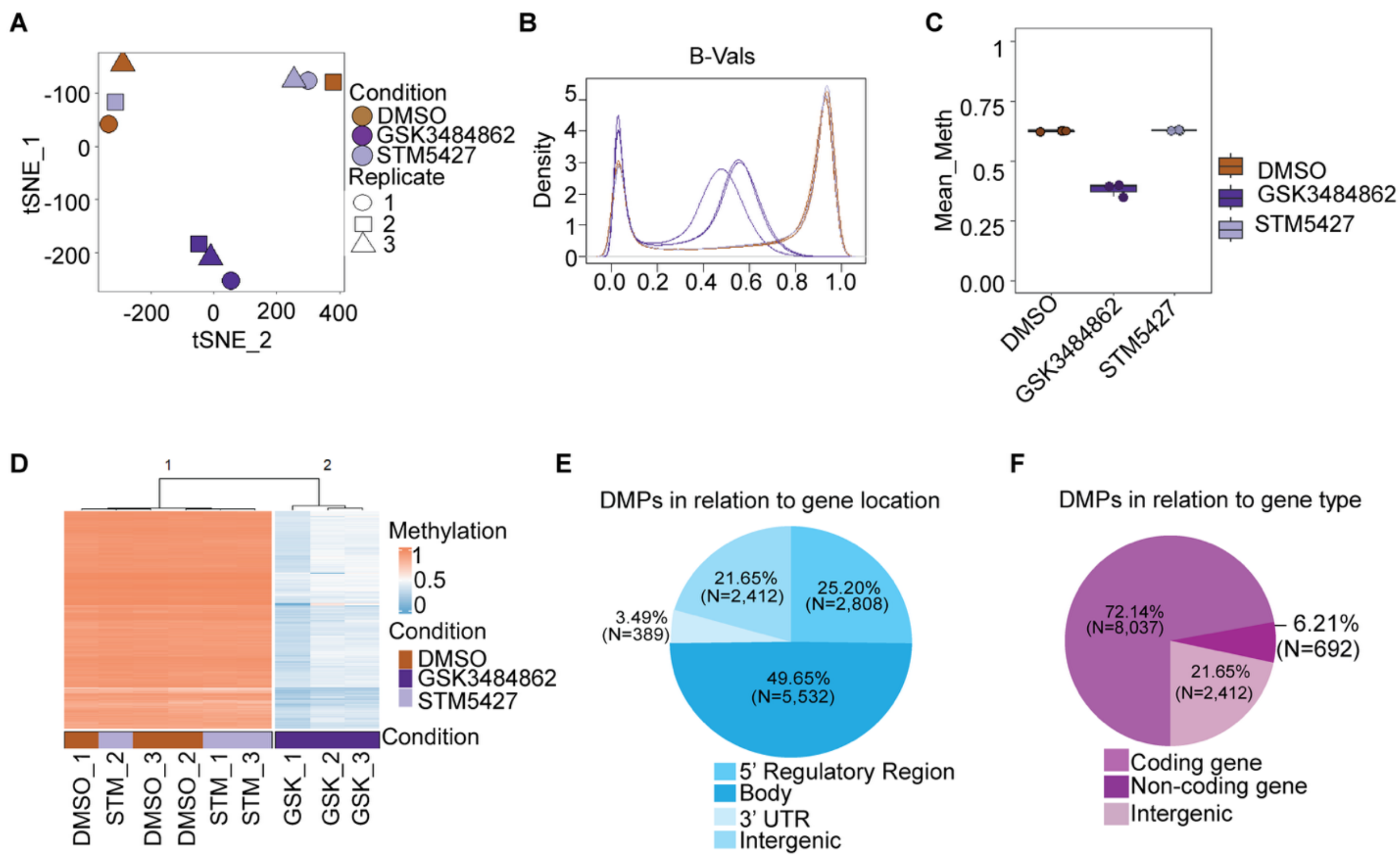

fig. S6. DNA methylation profiling of GSK3484862-treated OVCAR8 cells. (**A**) t-distributed Stochastic Neighbor Embedding (t-SNE) plot depicting the clustering of DMSO- (orange), GSK3484862- (violet), and STM2457-treated (light purple) experimental groups in three biological replicates. (**B**) Beta density plot showing DMSO control (orange), GSK3484862- (violet), and STM2457-treated (light purple) samples. **(C**) Scatter plot showing the mean methylation levels in DMSO-, GSK3484862-, and STM2457-treated OVCAR8 cells. (**D**) Heatmap displaying the unsupervised hierarchical clustering of the global DNA methylome in DMSO-, GSK3484862-, STM2457-treated groups. (**E** and **F**). Pie charts showing the distribution of differentially methylated probes in relation to gene location (**E**) and gene type (**F**). Data are presented from n = 3 independent biological experiments.

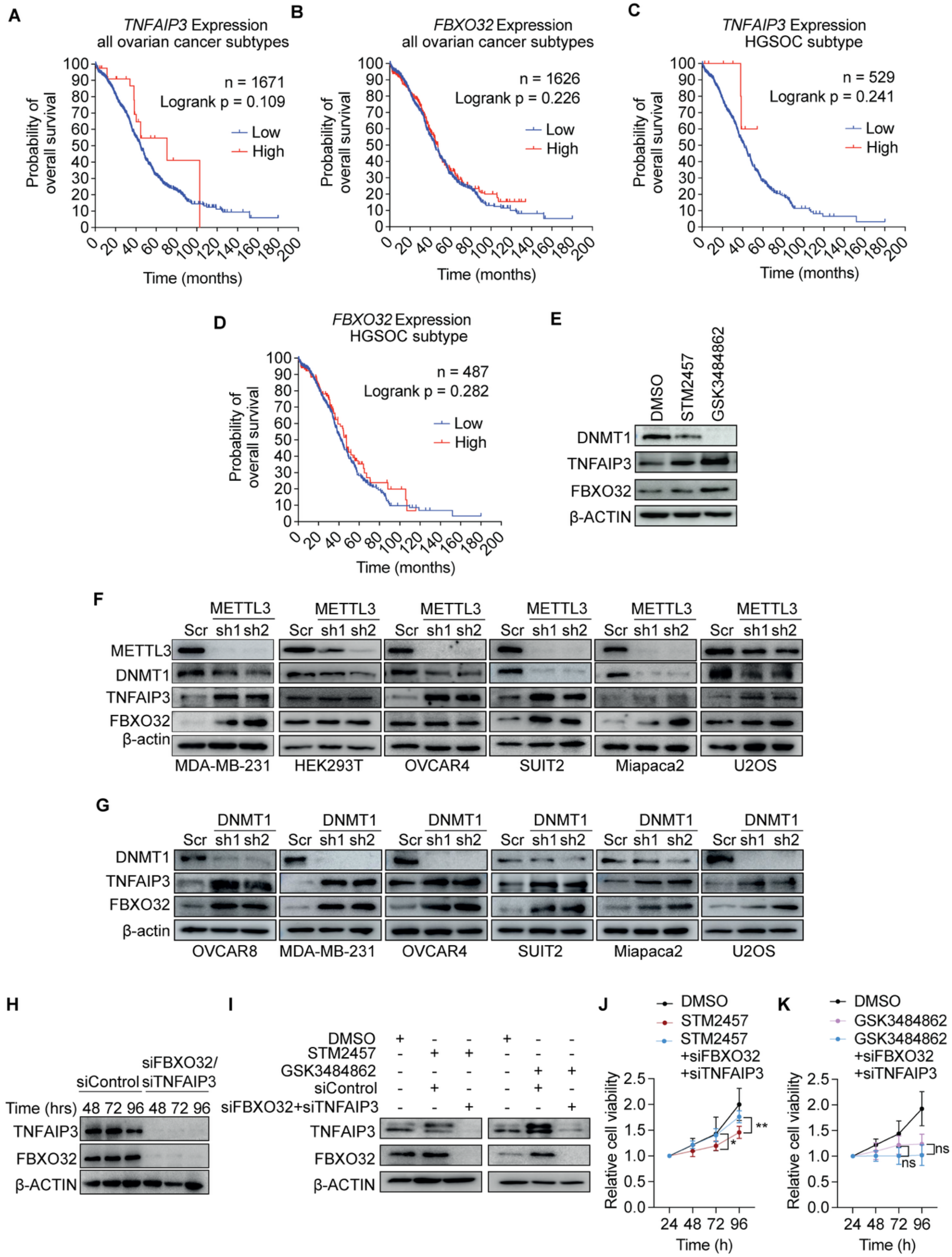

Figure legend on the next page

fig. S7. METTL3-DNMT1-dependent regulation of TNFAIP3 and FBXO32. (**A** to **D**) Overall survival analysis of TNFAIP3 (**A** and **C**) and FBXO32 (**B** and **D**) across all ovarian cancer subtypes (**A** and **B**) and in the HGSOC subtype (**C** and **D**) using TCGA data. (**E**) Western blot showing the expression of DNMT1, TNFAIP3, and FBXO32 following STM2457 and GSK3484862 treatment. (**F**) Immunoblots showing the effect of METTL3 knockdown on the expression of METTL3, DNMT1, TNFAIP3, and FBXO32 in MDA-MB-231, HEK293T, OVCAR4, SUIT2, MiaPaCa2, and U2OS cells. (**G**) Western blot showing the expression of DNMT1, TNFAIP3, and FBXO32 in DNMT1-depleted OVCAR8, MDA-MB-231, OVCAR4, SUIT2, MiaPaCa2, and U2OS cells. (**H**) Western blot showing knockdown efficiency of FBXO32 and TNFAIP3 at 48, 72, and 96 hours post-transfection with the respective siRNAs. (**I**) Western blot showing TNFAIP3 and FBXO32 protein levels in STM2457- (left) and GSK3484862-treated (right) OVCAR8 cells transfected with siControl or siRNAs targeting TNFAIP3 and FBXO32. (**J**) Line graph showing cell viability of DMSO- and STM2457-treated OVCAR8 cells with or without FBXO32 and TNFAIP3 co-silencing. (**K**) Line graph showing cell viability of DMSO- and GSK3484862-treated OVCAR8 cells with or without FBXO32 and TNFAIP3 co-silencing. For western blot analysis, β-ACTIN was used as the loading control. Two-tailed Student's *t*-test (**J** and **K)**. Data are mean ± s.d.; n = 2(**F** and **G**)**,** n=3 (**H** to **K**), independent biological experiments.

Data S1. (separate file)

data S1: RNA-seq analysis data for METTL3 KD cells compared to Scr control. Refer to the separate Excel file, table S1.xlsx

Data S2. (separate file)

data S2: RNA-seq analysis data for METTL14 KD cells compared to Scr control. Refer to the separate Excel file, table S2.xlsx

Data S3. (separate file)

data S3: MeRIP analysis data for OVCAR8 wild type. Refer to the separate Excel file, table S3.xlsx

Data S4. (separate file)

data S4: RNA-seq analysis data for GSK3484862-treated OVCAR8 cells compared to DMSO control. Refer to the separate Excel file, table S4.xlsx

Patient sample details		
**Patient id**	**Tissue type/ Histology subtype**	**Histology grade**
224b	Normal	NA
234d	Normal	NA
239d	Normal	NA
242d	Normal	NA
252d	Normal	NA
254d	Normal	NA
209a	Ovarian carcinoma, HGSOC	3
212a	Ovarian carcinoma, HGSOC	3
220s	Ovarian carcinoma, HGSOC	3
221a	Ovarian carcinoma, HGSOC	3
226a	Ovarian carcinoma, HGSOC	3
240a	Ovarian carcinoma, HGSOC	3
245a	Ovarian carcinoma, HGSOC	3
248a	Ovarian carcinoma, HGSOC	3
243a	Ovarian carcinoma, HGSOC	3
252a	Ovarian carcinoma, HGSOC	3
254a	Ovarian carcinoma, HGSOC	3

table S1: Details of ovarian patient samples used in the study.

**shRNAs**		
**Target Gene**	**Plasmid**	**Sequence 5´→ 3´**
Scramble	pLKO.1-puro-shScramble	CAACAAGATGAAGAGCACCAA
METTL3	pLKO.1-puro-sh*METTL3*_1	GCAAGTATGTTCACTATGAAA
	pLKO.1-puro-sh*METTL3*_2	CGTCAGTATCTTGGGCAAGTT
METTl14	pLKO.1-puro-sh*METTL14*_1	GCCGTGGACGAGAAAGAAATA
	pLKO.1-puro-sh*METTL14*_2	GCTAATGTTGACATTGACTTA
DNMT1	pLKO.1-puro-sh*DNMT1*_1	GCCCAATGAGACTGACATCAA
	pLKO.1-puro-sh*DNMT1*_2	GAGGTTCGCTTATCAACTAAT
**qPCR**		
**Gene**	**Forward primer 5´→ 3´**	**Reverse primer 5´→ 3´**
Mettl3	AACTGCAACGCATCATTCGG	TTGACACCAACCAAGCAGTG
METTL14	AGGGGTTGGACCTTGGAAGA	GAAGTCCCCGTCTGTGCTAC
β-actin	ACCAACTGGGACGACATGGAGAAG	TACGACCAGAGGCATACAGGGACA
DNMT1	GTGGCCCAGACTAGGAGGCG	CCGTGATGGTCCGGAAAGGAC

**Primers for SELECT of DNMT1 m^6A site**	
m^6A site_up	tagccagtaccgtagtgcgtgTCCACGTTCCCCACCCCCTG
m^6A site_down	CCCCACGTCCTGGAAGGAAAcagaggctgagtcgctgcat
Input_up	TagccagtaccgtagtgcgtgTTCCCCACCCCCTGTCCCCA
Input_down	GTCCTGGAAGGAAATTGGGAcagaggctgagtcgctgcat
qPCR_F for SELECT	ATGCAGCGACTCAGCCTCTG
qPCR_R for SELECT	TAGCCAGTACCGTAGTGCGTG

**Probes for Northern blotting**	**Sequence**
ITS1	CCTCGCCCTCCGGGCTCCGGGCTCCGTTAATGATC
ITS2	CTGCGAGGGAACCCCCAGCCGCGCA

**Luciferase reporter assay**	
psiCHECK2	All predicted m^6A sites in *Renilla* and *Firefly Luciferase* were mutated.
XhoI DNMT1_Exon8_F	GTCGAGTCAGGGCTCCTGAGATAATGA
NotI-DNMT1_Exon8_R	GCGGCCGCTTTCGATCACTAGCAAAACCCA
Sequencing primer	GCCTAAGATGTTCATCGAGTC
Gibson cloning primers	Lower case: Psicheck<br>Upper case: Dnmt1<br>Underline: m^6A mutation site<br>Fw1:<br>aggcgatcgctcgagTCAGGGCTCCTGAGATAA<br>Rev1:<br>TTTTGCTCTTTCAGGGTCTTCTGCAGGAAGCG<br>Forward 2:<br>CGCTTCCTGCAGAAGACCCTGAAAGAGCAAAA<br>Reverse 2:<br>GTTCCCCACCCCCTGGCCCCACGTCCTGGAAG<br>Forward 3:<br>CTTCCAGGACGTGGGGCCAGGGGGTGGGGAAC<br>Reverse 3:<br>gcggccagcggccgcTTTCGATCACTAGCAAAA

table S2: Primers, constructs and shRNAs sequences used in this study
